# TCM Constitution and the Risk of Hyperglycemia in Young and Middle‐Aged Adults: A Retrospective Cohort Study

**DOI:** 10.1155/jdr/5548887

**Published:** 2026-09-23

**Authors:** Jia-Hui Zhang, Yu-Han Zong, Fang-Li Li, Shi-Ping Hu, Meng-Ru Zhou, Xi-Huan Zhu, Hong-Fang Liu, Zhong-Yi Zhang, Huai-Yu Wang, Ji Wang, Qi Wang

**Affiliations:** ^1^ Wangqi Academy, National Institute of TCM Constitution and Preventive Medicine, Beijing University of Chinese Medicine, Beijing, China, bucm.edu.cn; ^2^ School of Traditional Chinese Medicine, Beijing University of Chinese Medicine, Beijing, China, bucm.edu.cn; ^3^ The Second School of Clinical Medicine, Guangzhou University of Chinese Medicine, Guangdong, Guangzhou, China, gzucm.edu.cn; ^4^ Department of Disease Prevention, Beijing University of Chinese Medicine Shenzhen Hospital, Guangdong, Shenzhen, China; ^5^ Department of Nephropathy, Beijing University of Chinese Medicine Affiliated Dongzhimen Hospital, Beijing, China; ^6^ Department of Endocrinology, Beijing University of Chinese Medicine Affiliated Dongzhimen Hospital, Beijing, China

**Keywords:** diabetes, hyperglycemia, risk predictor, TCM constitution

## Abstract

**Background:**

It is worth investigating whether the cluster of physical symptoms, body signs, and subjective feelings can be used to predict the risk of hyperglycemia, especially among the general young‐ and middle‐aged adults who irregularly receive lab tests.

**Methods:**

A retrospective cohort was established based on anonymous health check data from 2018 to 2023 in a single center. Hyperglycemia was identified if fasting blood glucose was ≥ 5.6 mmol/L and/or HbA1c ≥ 5.7%. Traditional Chinese medicine (TCM) body constitution was identified using China′s national standard questionnaire, which is a scheme of preventive medicine derived from traditional medicine, including nine types of constitution. Participants may have a single or mixed type of TCM constitution. Cox proportional hazards models were used to assess the association between TCM constitution and the risk of new‐onset hyperglycemia. Stratified analyses were performed by age, sex, and low‐density lipoprotein cholesterol levels.

**Results:**

Of the 8462 participants (37.46 ± 9.42 years) included, 2376 (28.08%) individuals developed hyperglycemia during the median follow‐up of 27.93 months. Blood stasis constitution (sublingual vein distension, pigmentation; HR = 2.30, 95% CI 1.38–3.82) and those mixed with others (HR = 1.72, 95% CI 1.18–2.52), qi stagnation constitution (sentimental, depressed; HR = 1.91, 95% CI 1.13–3.24) and those mixed with others (HR = 1.87, 95% CI 1.24–2.83), phlegm‐dampness constitution (soft/fat belly, loose and sticky stool; HR = 1.65, 95% CI 1.09–2.49), yang‐deficient constitution (cold‐sensitive; HR = 2.27, 95% CI 1.13–4.57), and damp‐heat constitution (halitosis, prone to acne; HR = 1.48, 95% CI 1.03–3.14) showed significant association with the increased risk of hyperglycemia in the general population. With the exception of yang‐deficient constitution (insufficient sample size), all other TCM constitutions showed significantly higher association with hyperglycemia in adults aged 18–44 years, but not in those aged ≥ 45 years.

**Conclusion:**

Assessment based on a TCM constitutional questionnaire can serve as a risk indicator of the risk of hyperglycemia in the general population, particularly among young adults.

## 1. Introduction

Diabetes and prediabetes have emerged as major health challenges worldwide. In 2021, the population with impaired fasting glucose (IFG) reached 298 million among adults aged 20–79 years, and this number is projected to increase to 414 million by 2045 [1]. Furthermore, Type 2 diabetes is increasingly diagnosed at younger ages [2], with young‐onset cases more likely to result in severe complications and early death [3]. Taken together, the early risk prediction of hyperglycemia for young adults is important.

According to the *American Diabetes Association (ADA) Standards of Care*, the ADA risk test is used to assess diabetes risk, incorporating conventional factors such as age, sex, medical history, family history, hypertension, physical activity, and weight [4]. Although this established methodology offers a standardized approach to risk assessment, it is inadequate to identify high‐risk populations of hyperglycemia without conventional risk factors. Aiming at risk evaluation and prevention of hyperglycemia as early as possible, it is worth exploring a noninvasive and easy‐to‐use approach to evaluate the risk of hyperglycemia, especially for the population not covered by the ADA risk test.

Traditional Chinese medicine (TCM) body constitution can be regarded as a systematic approach to assessing physical phenotype, originally derived from TCM and established based on two waves of national surveys [5, 6]. Physical phenotype, which can be identified as a cluster of body signs, symptoms, and subjective feelings, was generally considered to be accompanied by nonspecific symptoms of the primary chronic disease or as the manifestation of subhealth status [5, 7–10]. Values of TCM constitution in the evaluation of subhealth or preclinical status, or as a risk monitor of the disease, were underexplored. Theoretically, a specific cluster of body signs and symptoms was the manifestation of some physical and/or psychological abnormalities, while these abnormalities may not meet any known diagnostic criteria yet. It is reasonable to hypothesize that a specific TCM constitution might be the very early signal of the disease and may be used as a risk indicator. The pattern of body signs, symptoms, and subjective feelings was scored using the standard questionnaire so that nine types of TCM constitution were identified [11–13]. Each TCM constitution—the physical phenotype—was named according to the representative abnormality defined in TCM [11, 12]. Given its value as a risk monitor of multiple diseases, TCM constitution has been included in the National Basic Public Health Service Projects of China since 2011 [14, 15].

Previous cross‐sectional studies demonstrated that phlegm‐dampness constitution exhibits a significant correlation with diabetes, dyslipidemia, hyperuricemia, and metabolic dysfunction‐associated fatty liver disease [16–21]. However, in the general population, many people have two or more types of TCM constitution, such as the coexistence of phlegm‐dampness and qi‐deficient constitution. In sum, it is worthwhile to conduct a longitudinal cohort study to examine the effects of both single‐ and mixed‐biased constitutional types on the risk of hyperglycemia.

Hence, the present study established a retrospective cohort using health check data of the general population to evaluate whether the physical phenotype evaluated by TCM constitution may serve as a risk indicator of new‐onset hyperglycemia to assist risk stratification.

## 2. Methods

### 2.1. Study Population

It was a retrospective cohort study using anonymous health check data from the Affiliated Hospital of Beijing University of Chinese Medicine from 2018 to 2023. A total of 10,862 participants aged ≥ 18 years who received the assessment of TCM constitution using the standard questionnaire were initially included. Exclusion criteria were as follows: (i) received a health check only once (*n* = 115) and (ii) fasting blood glucose (FBG) < 3.9 or ≥ 5.6 mmol/L or HbA1c ≥ 5.7% at baseline (*n* = 2285). Ultimately, a total of 8462 participants were eligible for the present study.

The present study was approved by the Institutional Review Board of the Ethics Committee of Shenzhen Hospital affiliated with Beijing University of Chinese Medicine (KY‐2024‐159).

### 2.2. TCM Constitution

The TCM constitution was evaluated using the questionnaire from the Chinese industry standard Classification and Determination of Constitution in TCM (ZYYXH/T157‐2009) [11–13] (Table S1). The assessment was primarily self‐administered with guidance from physicians trained in a standardized protocol, and only the final TCM constitution classification was recorded in the anonymized dataset. Nine types of TCM constitution were recognized using the questionnaire, including one balanced constitution (with a relatively healthy and energetic status) and eight types of bias constitution: qi‐deficient constitution (characterized as having low voice, often feeling exhausted and tired), yang‐deficient constitution (characterized as sensitive to cold, having cold hands and feet), yin‐deficient constitution (characterized as having night sweats, internal heat, and dryness), phlegm‐dampness constitution (characterized as having oily face, sticky mouth, and feeling heaviness), damp‐heat constitution (characterized as easy to have acne, halitosis, and being irritable), blood stasis constitution (characterized as engorgement of tongue sublingual vein, easy to have bruises, and nonspecific pain), qi stagnation constitution (characterized as sentimental and easy to have mental disorders), and intrinsic/allergic constitution (characterized as suffering allergies) [12]. Two or more types of bias constitution can coexist in the same person [5]. Mixed‐biased constitutions were analyzed as composite categories (Table S1). Ultimately, seven types of mixed bias constitution were included, which were yang‐deficient mixed with others, qi‐deficient mixed with others, qi stagnation mixed with others, phlegm‐dampness mixed with others, damp‐heat mixed with others, blood stasis mixed with others, and yin‐deficient mixed with others (Table S2). In the present analyses, types of TCM constitution showing small sample size (*n* ≤ 15) were excluded.

### 2.3. New‐Onset Hyperglycemia

The follow‐up duration was 27.93 (14.80, 41.13) months. According to the *ADA Standards of Care in Diabetes 2025*, participants with normal levels of blood glucose at baseline but occurred diabetes mellitus (FBG ≥ 7.0 mmol/L and/or HbA1c ≥ 6.5%) or prediabetes (FBG 5.6–6.9 mmol/L and/or HbA1c 5.7%–6.4%) during the follow‐up duration were defined as new‐onset hyperglycemia [4].

### 2.4. Covariates

Age and sex were recorded. Based on a commonly used age stratification in epidemiological studies [22, 23], participants were categorized into 18–44 and ≥ 45 years in the primary age‐stratified analyses. Total cholesterol (TC), triglycerides (TG), low‐density lipoprotein cholesterol (LDL‐C), high‐density lipoprotein cholesterol (HDL‐C), uric acid (UA), and serum creatinine (SCr) at baseline were recorded. Body mass index (BMI) was calculated by body weight (kilograms) divided by the square of height (meters) and classified into underweight (< 18.5 kg/m^2^), normal (18.5–24 kg/m^2^), overweight (24–28 kg/m^2^), and obesity (≥ 28 kg/m^2^) [24]. Hypertension was defined as systolic blood pressure (SBP) ≥ 140 mmHg or diastolic blood pressure (DBP) ≥ 90 mmHg [25].

### 2.5. Statistical Analyses

Baseline characteristics were presented and compared between groups stratified by having new‐onset hyperglycemia or not. Continuous variables were presented as mean ± standard deviation (SD) for normally distributed data and medians (interquartile range, IQR) for non‐normally distributed data. Categorical variables were presented as percentages. Group comparisons were performed using Student′s *t*‐test for normally distributed continuous variables, the rank‐sum test for non‐normally distributed continuous variables, and the chi‐square test for categorical variables, respectively (Tables 1 and 2).

**Table 1:** Baseline characteristics.

Variables	Overall (*n* = 8462)	New‐onset hyperglycemia (*F* *B* *G* ≥ 5.6 mmol/L or *H* *b* *A*1*c* ≥ 5.7%)		*p*value
		No (*n* = 6086)	Yes (*n* = 2376)	
Age (mean ± SD, years)	37.46 ± 9.42	36.17 ± 9.04	40.75 ± 9.59	< 0.001
Age group (*n*, %)				
18–44 years	6492 (76.72)	4951 (81.35)	1541 (64.86)	< 0.001
≥ 45 years	1970 (23.28)	1135 (18.65)	835 (35.14)	
Sex (*n*, %)				
Male	4464 (52.75)	2918 (47.95)	1546 (65.07)	< 0.001
Female	3998 (47.25)	3168 (52.05)	830 (34.93)	
BMI (mean ± SD, kg/m^2^)	22.97 ± 3.33	22.55 ± 3.27	24.07 ± 3.25	< 0.001
BMI group (*n*, %)				
Underweight	549 (7.46)	494 (9.30)	55 (2.69)	< 0.001
Normal	4168 (56.66)	3165 (59.57)	1003 (49.09)	
Overweight	2107 (28.64)	1355 (25.50)	752 (36.81)	
Obesity	532 (7.23)	299 (5.63)	233 (11.40)	
SBP (mean ± SD, mmHg)	116.30 ± 13.05	115.30 ± 12.68	118.87 ± 13.62	< 0.001
DBP (mean ± SD, mmHg)	71.67 ± 10.28	70.87 ± 10.00	73.74 ± 10.70	0.008
Hypertension (*n*, %)				
Yes	524 (6.88)	315 (5.74)	209 (9.78)	< 0.001
No	7097 (93.12)	5169 (94.26)	1928 (90.22)	
TC (mean ± SD, mmol/L)	4.93 ± 0.98	4.86 ± 0.96	5.11 ± 0.99	< 0.001
TG (median [IQR], mmol/L)	1.10 (0.79, 1.61)	1.03 (0.75, 1.50)	1.30 (0.93, 1.85)	< 0.001
LDL‐C (mean ± SD, mmol/L)	3.27 ± 0.76	3.21 ± 0.76	3.41 ± 0.75	< 0.001
HDL‐C (mean ± SD, mmol/L)	1.39 ± 0.32	1.41 ± 0.32	1.33 ± 0.31	< 0.001
UA (median [IQR], *μ*mol/L)	359.90 (291.40, 439.20)	346.90 (281.50, 429.90)	392.55 (322.15, 458.45)	< 0.001
SCr (median [IQR], *μ*mol/L)	74.10 (61.00, 86.75)	72.00 (60.00, 85.70)	78.64 (64.30, 88.70)	< 0.001
FBG (median [IQR], mmol/L)	4.81 (4.53, 5.08)	4.74 (4.48, 5.01)	4.97 (4.69, 5.24)	< 0.001
HbA1c (median [IQR], %)	5.40 (5.10, 5.50)	5.30 (5.10, 5.50)	5.40 (5.20, 5.60)	< 0.001
Follow‐up duration (median [IQR], months)	27.93 (14.80, 41.13)	29.52 (16.50, 44.13)	25.83 (13.33, 36.82)	< 0.001

Abbreviations: BMI, body mass index; DBP, diastolic blood pressure; FBG, fasting blood glucose; HbA1c, hemoglobin A1c; HDL‐C, high‐density lipoprotein cholesterol; LDL‐C, low‐density lipoprotein cholesterol; SBP, systolic blood pressure; SCr, serum creatinine; TC, total cholesterol; TG, triglycerides; UA, uric acid.

**Table 2:** Distribution of TCM constitution in population with or without new‐onset hyperglycemia.

TCM constitution	Overall (*n* = 8461)	New‐onset hyperglycemia		*p*value
		No (*n* = 6085)	Yes (*n* = 2376)	
Balanced constitution (*n*, %)	362 (4.28)	311 (5.11)	51 (2.15)	< 0.001
Single‐biased constitution (*n*, %)				
Yang‐deficient	71 (16.40)	49 (13.61)	22 (30.14)	0.001
Yin‐deficient	252 (41.04)	193 (38.29)	59 (53.64)	0.003
Qi‐deficient	384 (51.47)	286 (47.91)	98 (65.77)	< 0.001
Qi stagnation	294 (44.82)	210 (40.31)	84 (62.22)	< 0.001
Phlegm‐dampness	870 (70.62)	634 (67.09)	236 (82.23)	< 0.001
Damp‐heat	2521 (87.44)	1839 (85.53)	682 (93.04)	< 0.001
Blood stasis	294 (44.82)	201 (39.26)	93 (64.58)	< 0.001
Mixed‐biased constitution (*n*, %)				
Yang‐deficient mixed	126 (25.82)	91 (22.64)	35 (40.70)	0.001
Qi‐deficient mixed	737 (67.06)	535 (63.24)	202 (79.84)	< 0.001
Qi stagnation mixed	898 (71.27)	588 (65.41)	310 (85.87)	< 0.001
Phlegm‐dampness mixed	1621 (81.74)	1137 (78.52)	484 (90.47)	< 0.001
Damp‐heat mixed	1410 (79.57)	968 (75.68)	442 (89.66)	< 0.001
Blood stasis mixed	1597 (81.52)	1098 (77.93)	499 (90.73)	< 0.001
Yin‐deficient mixed	437 (54.69)	307 (49.68)	130 (71.82)	< 0.001

*Note:* Intrinsic/allergic constitution was excluded because of the sample size (*n* = 1).

**Figure 1:**
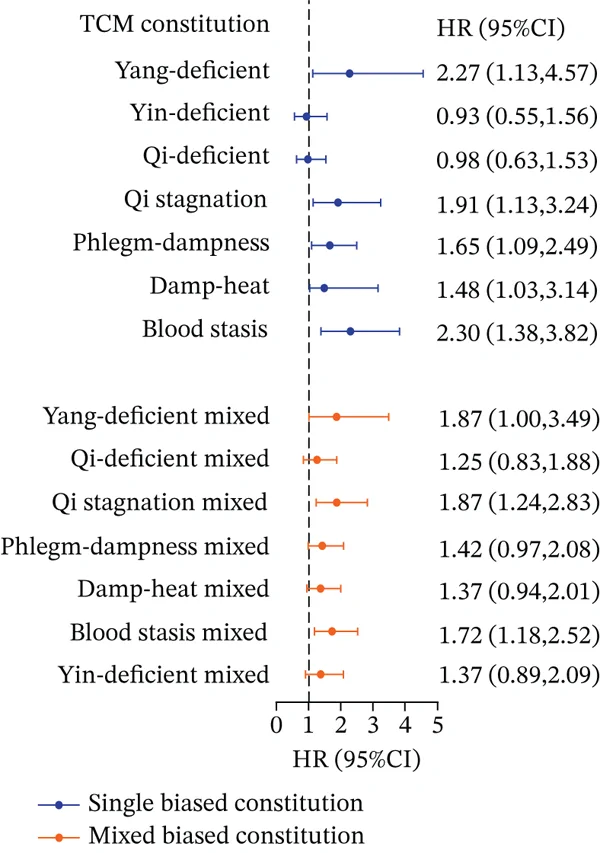
Association between TCM constitution and risk of hyperglycemia. Age, sex, BMI, SBP, TC, TG, LDL‐C, UA, and SCr were adjusted. HR, hazard ratio; 95% CI, 95% confidence interval.

Cox proportional hazards regression models were employed to assess the association between TCM constitution and the risk of new‐onset hyperglycemia. Results were presented using hazard ratios (HRs) with 95% confidence intervals (CIs). Confounders including sex, age, BMI, SBP, TC, TG, LDL‐C, UA, and SCr were stepwise adjusted (Figure 1).

**Figure 2:**
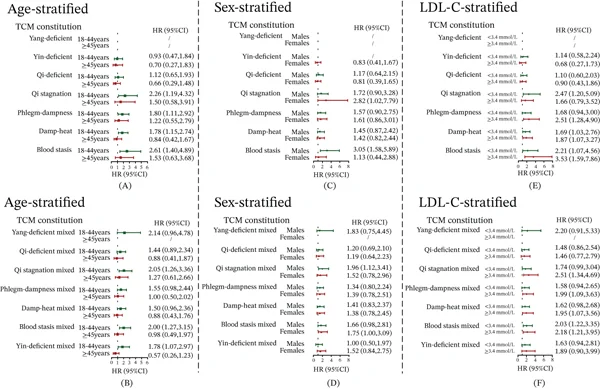
Association between TCM constitution and risk of hyperglycemia, age‐, sex‐, and LDL‐C‐stratified analyses. (A,B) Association between TCM constitution and risk of hyperglycemia stratified by age (18–44 and ≥ 45 years). Sex, BMI, SBP, TC, TG, LDL‐C, UA, and SCr were adjusted. (C,D) Association between TCM constitution and risk of hyperglycemia stratified by sex (male and female). Age, BMI, SBP, TC, TG, LDL‐C, UA, and SCr were adjusted. (E,F) Association between TCM constitution and risk of hyperglycemia stratified by LDL‐C (≤ 3.4 and > 3.4 mmol/L). Age, sex, BMI, SBP, TC, TG, UA, and SCr were adjusted. The symbol “/” represented that the bias constitution was excluded because of the sample size (*n* ≤ 15).

Given the strong influence of sex, age, and LDL‐C on the association between TCM constitution and the risk of hyperglycemia, stratified analyses were further conducted (Figure 2, Table 3, and Table S3). To assess whether the association between TCM constitution and the risk of hyperglycemia varies by age, a constitution × age interaction term was included in the Cox regression models (Table S4).

**Table 3:** Association between TCM constitution and risk of hyperglycemia cross‐stratified by sex (male and female) and age (18–44 and ≥ 45 years).

TCM constitution	Male						Female					
	18–44 years			≥ 45 years			18–44 years			≥ 45 years		
	*N*	Fully adjusted HR (95% CI)	*p*value	*N*	Fully adjusted HR (95% CI)	*p*value	*N*	Fully adjusted HR (95% CI)	*p*value	*N*	Fully adjusted HR (95% CI)	*p*value
Balanced	13	Ref.	Ref.	3	Ref.	Ref.	10	Ref.	Ref.	6	Ref.	Ref.
Yang‐deficient	5	/	/	6	/	/	4	/	/	4	/	/
Yin‐deficient	2	/	/	5	/	/	14	/	/	26	0.44 (0.14, 1.43)	0.174
Qi‐deficient	25	1.17 (0.56, 2.41)	0.678	22	1.35 (0.33, 5.33)	0.673	11	/	/	18	0.21 (0.06, 0.72)	0.013
Qi stagnation	34	1.64 (0.76, 3.54)	0.209	21	3.29 (0.74, 14.69)	0.118	10	/	/	4	/	/
Phlegm‐dampness	87	1.53 (0.81, 2.86)	0.187	35	1.84 (0.50, 6.79)	0.363	44	1.76 (0.80, 3.86)	0.160	23	0.84 (0.30, 2.35)	0.743
Damp‐heat	251	1.44 (0.81, 2.55)	0.209	118	1.67 (0.52, 5.40)	0.392	132	2.25 (1.14, 4.47)	0.020	56	0.53 (0.21, 1.32)	0.173
Blood stasis	43	2.93 (1.39, 6.17)	0.005	29	4.42 (0.92, 21.24)	0.063	6	/	/	5	/	/
Yang‐deficient mixed	9	/	/	7	/	/	8	/	/	3	/	/
Qi‐deficient mixed	76	1.13 (0.60, 2.11)	0.701	34	1.75 (0.48, 6.37)	0.394	32	1.85 (0.86, 3.96)	0.114	18	0.50 (0.15, 1.65)	0.254
Qi stagnation mixed	145	1.78 (0.95, 3.33)	0.070	56	2.42 (0.72, 8.12)	0.154	35	2.10 (0.90, 4.91)	0.087	23	0.89 (0.28, 2.87)	0.845
Phlegm‐dampness mixed	222	1.29 (0.72, 2.30)	0.387	88	1.69 (0.52, 5.52)	0.386	51	1.76 (0.83, 3.72)	0.138	30	0.87 (0.30, 2.50)	0.796
Damp‐heat mixed	164	1.38 (0.76, 2.48)	0.286	87	1.66 (0.51, 5.42)	0.399	61	1.79 (0.86, 3.72)	0.118	41	0.49 (0.19, 1.24)	0.131
Blood stasis mixed	169	1.67 (0.92, 3.03)	0.093	93	1.90 (0.58, 6.18)	0.286	94	2.48 (1.21, 5.11)	0.013	54	0.46 (0.18, 1.13)	0.090
Yin‐deficient mixed	21	1.00 (0.45, 2.18)	0.990	15	/	/	45	2.32 (1.10, 4.91)	0.027	27	0.55 (0.20, 1.55)	0.258

*Note:* BMI, SBP, TC, TG, LDL‐C, UA, and SCr were adjusted. The symbol “/” represented that the bias constitution was excluded because of the sample size (*n* ≤ 15).

To assess the robustness of the findings, several sensitivity analyses were conducted. Additional age‐stratified analyses were conducted by referencing the ADA diabetes risk score, which begins assigning higher risk weights at age 40 [4], and participants were recategorized into 18–39 and ≥ 40 years (Table S5). Given that constitution‐related susceptibility may require sufficient exposure time to manifest clinically, follow‐up‐stratified analyses were performed to assess whether a single baseline constitution assessment predicted hyperglycemia over longer versus shorter periods. Two complementary cutoffs were used: 24 months (approximately 2 years, a conventional follow‐up benchmark) and 30 months (approximating the rounded cohort median of 27.93 months). Participants with < 6 months of follow‐up were excluded from the 30‐month analysis to reduce potential confounding by subclinical disease at baseline (*n* = 8) (Tables S6 and S7). Given that individuals with different constitution types may differ systematically in baseline characteristics, the sensitivity analyses were performed in participants with matched age, sex, and BMI using propensity score matching (PSM) (Tables S8 and S9). To assess whether the composite endpoint (both prediabetes and diabetes) obscures constitutional effects on distinct stages of glycemic deterioration, sensitivity analyses were conducted separately using new‐onset prediabetes as the outcome (Tables S10 and S11).

All *p* values were two‐tailed, and a *p* value < 0.05 was defined as statistically significant. All analyses were conducted using Stata Version 18.0 (Stata Corp LP, College Station, TX, United States).

## 3. Results

### 3.1. Baseline Characteristics

A total of 8462 participants aged 37.46 ± 9.42 years were included. Among them, 52.75% were males. With a follow‐up of 27.93 (14.80, 41.13) months, 2376 (28.08%) individuals developed new‐onset hyperglycemia. Compared with individuals who kept normal levels of blood glucose, higher levels of UA and SCr, and higher proportions of males, older adults, hypertension, and overweight/obesity were observed in those with new‐onset hyperglycemia (*p* < 0.05) (Table 1).

Compared with adults with a balanced constitution, significantly higher proportions of new‐onset hyperglycemia were observed in adults with yin‐deficient constitution, qi stagnation constitution, phlegm‐dampness constitution, damp‐heat constitution, blood stasis constitution, and all mixed‐biased constitution (Table 2).

### 3.2. TCM Constitution and the Risk of New‐Onset Hyperglycemia

After adjustment for confounders, blood stasis and yang‐deficient were associated with over twofold increased risk of hyperglycemia, followed by qi stagnation constitution (HR = 1.91, 95% CI 1.13–3.24), phlegm‐dampness constitution (HR = 1.65, 95% CI 1.09–2.49), and damp‐heat constitution (HR = 1.48, 95% CI 1.03–3.14). Regarding the mixed constitution, only the mixed constitution containing qi stagnation and blood stasis showed a significant association with the increased risk of hyperglycemia (Figure 1).

In stepwise Cox proportional hazards regression analyses, we observed that the association between seven of 14 types of biased constitution and the risk of hyperglycemia reversed upon adjustment for age, sex, or LDL‐C (Table S3). Therefore, age‐, sex‐, and LDL‐C‐stratified analyses were further performed (Figure 2 and Table 3).

### 3.3. Age‐ and Sex‐Stratified Analyses

In the fully adjusted Cox models, constitution × age interaction terms were not significant for the majority of biased constitution types (*p* > 0.05). One exception was blood stasis mixed with other constitutions, which showed a significant interaction with age (*p* = 0.047), with the association attenuating in older participants (Table S4).

TCM constitution was the risk predictor of the onset of hyperglycemia in the younger adults. In detail, qi stagnation constitution and those mixed with other constitutions, blood stasis constitution and those mixed with other constitutions, yang‐deficient constitution, phlegm‐dampness constitution, damp‐heat constitution, and yin‐deficient mixed with others were risk predictors of hyperglycemia in adults aged 18–44 years, but not in those aged ≥ 45 years (Figure 2A,B).

Significant association between yang‐deficient constitution, blood stasis constitution, qi stagnation mixed with others, and the increased risk of hyperglycemia was only observed in males, but not in females. The qi stagnation constitution showed a significant association with the risk of new‐onset hyperglycemia in females, but not in males (Figure 2C,D).

To perform cross‐stratified analyses, age‐ and sex‐combined categories were defined as male or female aged 18–44 or aged ≥ 45 years. A significant association between blood stasis constitution and the increased risk of hyperglycemia was observed in males aged 18–44 years. Yin‐deficient mixed with other constitution, blood stasis mixed with other constitution, and damp‐heat constitution showed a significant association with the risk of new‐onset hyperglycemia in females aged 18–44 years. Qi‐deficient constitution was significantly associated with a lower risk of new‐onset hyperglycemia in females aged ≥ 45 years (Table 3).

### 3.4. LDL‐C‐Stratified Analyses

A significant association between qi stagnation constitution and the increased risk of hyperglycemia was only observed in adults with normal LDL. Phlegm‐dampness constitution and those mixed with other constitutions, qi stagnation mixed with others, and damp‐heat mixed with others were the risk predictors of new‐onset hyperglycemia in the elevated LDL‐C group, but not in the normal LDL‐C group (Figure 2E,F).

### 3.5. Sensitivity Analyses

Similar results were observed in the age‐stratified analyses using 40 years as the age cut‐off. Qi stagnation constitution and those mixed with other constitutions, blood stasis constitution and those mixed with other constitutions, and damp‐heat constitution were risk predictors of hyperglycemia in adults aged 18–39 years, but not in those aged ≥ 40 years (Table S5).

Qi stagnation constitution and those mixed with other constitutions, blood stasis constitution and those mixed with other constitutions, damp‐heat constitution, and yang‐deficient mixed others showed a significant association with the risk of new‐onset hyperglycemia among participants with ≥ 24 months of follow‐up, but not among participants with < 24 months of follow‐up (Table S6). The qi stagnation constitution and those mixed with other constitutions and the blood stasis constitution and those mixed with other constitutions were risk predictors of hyperglycemia among participants with ≥ 30 months of follow‐up, but not among participants with 6–30 months of follow‐up (Table S7).

In the PSM analyses, all constitution types significantly associated with hyperglycemia in the main analyses showed consistent directions of effect. Phlegm‐dampness constitution, blood stasis constitution, and qi stagnation mixed with others remained statistically significant after matching, which were consistent with the main analyses (Table S9).

In the sensitivity analyses restricted to new‐onset prediabetes (*n* = 2332), the types of TCM constitution significantly associated with hyperglycemia in the main analyses all remained significantly associated with prediabetes (Tables S10 and S11).

## 4. Discussion

Based on the retrospective cohort of the general population, the present study demonstrated that TCM constitution, which was a method of physical phenotyping derived from traditional medicine, can serve as a risk indicator of the onset of hyperglycemia, especially in adults younger than 45 years. The risk constitution, including phlegm‐dampness constitution, characterized as having a chubby physique, loose and sticky stool, and feeling heaviness; damp‐heat constitution, characterized as having acne and being irritable; yang‐deficient constitution, characterized as cold intolerance; blood stasis constitution, characterized as subcutaneous bruising and fixed stabbing pain; and qi stagnation constitution, characterized as a sentimental personality, can be easily identified using the standard questionnaire of TCM constitution. In contrast to the conventional monitoring of hyperglycemia using lab tests, noninvasive methods showed feasibility and clinical implications for the young general population.

Noninvasive and cost‐efficient methods are always needed for risk prediction. The ADA risk test evaluates the risk of diabetes using a questionnaire including age, sex, medical history, family genetic history, hypertension, physical activity, and weight category [4]. This questionnaire emphasized the risk effect of advanced age and conventional risk factors of diabetes but did not consider physical manifestations. There were issues to be solved. First, given the rising incidence of diabetes in the young population, as well as the highlight of early action in diabetes prevention, exploration of risk predictors of hyperglycemia for young adults was necessary [2, 26]. Second, the high‐risk population of hyperglycemia in the general population without conventional risk factors for diabetes should not be neglected. Third, physical manifestations were likely to be a signal of pathogenesis or worse health status [5]. Actually, identifying physical phenotype using questionnaires has been a conventional method to target the high‐risk population for diseases and outcomes, whereas a better understanding of physical phenotype has been obtained in older adults and patients with diagnosed diseases rather than in the general young population [5, 7–9]. For instance, the symptom of swallowing‐related cough was demonstrated to be a signal of worse physical and mental health, as well as a risk factor for aspiration pneumonia [27]. The condition of frailty is a kind of physical phenotype and was demonstrated to be a risk factor for multiple diseases and adverse outcomes in older adults [28–30]. Thus, it is worth investigating the physical phenotype related to the risk of hyperglycemia in the general population. The TCM constitution is a method derived from traditional medicine to identify the stable physical phenotype in the general population using a standard questionnaire. Previous studies demonstrated the association between phlegm‐dampness constitution and diabetes [17]. According to the present results, several types of constitution showed a significant association with the increased risk of new‐onset hyperglycemia, especially in adults aged younger than 45 years. These findings indicated that TCM constitution may serve as a noninvasive risk predictor of hyperglycemia and supported its potential role in risk stratification in the general young population.

Several sensitivity analyses supported the robustness of these findings. After using 40 years as the age cut‐off, the association remained significant in younger adults (18–39 years). The findings of PSM analyses demonstrated that they were not driven by imbalances in the general baseline characteristics. The follow‐up‐stratified analyses further suggested that TCM constitution holds long‐term predictive value for hyperglycemia, consistent with the interpretation that constitution reflects underlying disease susceptibility requiring prolonged exposure to manifest clinically [6]. Results remained unchanged when participants who developed diabetes were excluded. Taken together, these analyses indicated that TCM constitution may have earlier predictive capacity for the risk of new‐onset hyperglycemia, particularly among young adults.

Regarding the mechanism by which types of TCM constitution induce hyperglycemia, previous studies demonstrated that phlegm‐dampness constitution changed gut microbial composition, inflammatory responses, and gene expression, leading to insulin resistance and metabolic disorder, thereby driving hyperglycemia [31–35]. In addition, the gut microbial composition of a population with damp‐heat constitution exhibits significant alteration, resulting in low‐grade inflammation and obesity, subsequently contributing to insulin resistance and even hyperglycemia [36–38]. Previous studies found that, compared with a balanced constitution, a yang‐deficient constitution showed significant differences in multiple metabolic indexes such as BMI, DBP, FBG, UA, and TG [32]. Blood stasis is characterized by systemic hemodynamic changes, and individuals suffering from blood stasis may have the deformability of red blood cells deteriorate, abnormal platelet function, increased blood viscosity, and an inflammatory response [39–41]. Accumulating evidence indicates that hemodynamic and microvascular abnormalities may be potential mechanisms of insulin resistance and hyperglycemia [42, 43]. Previous studies have supported that qi stagnation constitution is closely associated with psychological disorders (e.g., anxiety/depression) [44–46]. Chronic psychological stress can promote gluconeogenesis and inhibit insulin secretion through the hypothalamic–pituitary–adrenal (HPA) axis [47, 48]. Meanwhile, anxiety has also been proven to be positively correlated with diabetes [46]. Taken together, the risk effects of TCM constitution on the increased risk of hyperglycemia can be partly explained by the existing pathophysiological evidence, although more grounded and direct mechanisms underlying these types of TCM constitution remain to be investigated.

The present study has several limitations. First, geographical bias exists in the distribution of TCM constitution. Subject to the nature of a single‐center study, the association between TCM constitution and hyperglycemia in other regions with different climates, ethnics, and lifestyles should be further validated. Second, due to the insufficient sample size of the reference group, the results of cross‐stratified analyses were taken with caution. Third, due to the relatively short follow‐up duration (median 27.93 months), the number of new‐onset diabetes events was limited (*n* = 44), which restricted the investigation of the TCM constitution on the risk of diabetes. Fourth, data on lifestyles (e.g., dietary patterns and physical activity), medication history (e.g., antihypertensive or lipid‐lowering drugs), family history of diabetes, and environmental exposures were not available in the present study. The influence of these factors on TCM constitution and risk of hyperglycemia was not assessed, and it should be further investigated in the future. Fifth, the anonymized dataset retained only the final constitution classification; individual dimension scores were not accessible, precluding analyses that distinguish primary from secondary constitutions. Future studies with access to raw dimension scores are warranted to examine whether the observed associations differ by constitutional primacy.

## 5. Conclusion

The present study constructed a retrospective cohort study and demonstrated that TCM constitution, a method easy to assess using a standard questionnaire, can be used as a noninvasive, accurate, and cost‐saving risk predictor of hyperglycemia in the general population, especially in adults younger than 45 years. These findings show clinical implications of TCM constitution in the prevention of hyperglycemia.

## Author Contributions

J‐H.Z.: conceptualization, methodology, formal analyses, investigation, visualization, writing—original draft, and writing—review and editing. Y‐H.Z.: methodology and review and editing. F‐L.L.: data curation and review and editing. S‐P.H.: data curation and review and editing. M‐R.Z.: methodology and review and editing. X‐H.Z.: methodology and review and editing. H‐F.L.: data curation and review and editing. Z‐Y.Z.: review and editing. H‐Y.W.: conceptualization, methodology, funding acquisition, investigation, project administration, supervision, and writing—review and editing. J.W.: supervision, funding acquisition, and review and editing. Q.W.: funding acquisition and resources.

## Funding

This study was funded by the National Natural Science Foundation of China (10.13039/501100001809) (T2341006), the National Key R&D Program of China (2024YFC3506300 and 2024YFC3506301), the Fundamental and Interdisciplinary Disciplines Breakthrough Plan of the Ministry of Education of China (JYB2025XDXM612), the National Natural Science Foundation of Beijing (L232118), the Discipline Team Construction Project of Beijing University of Chinese Medicine (BUCM‐XKTD20250201), the Beijing University of Chinese Medicine Basic Research Operating Funds (2023‐JYB‐JBZD‐023), the Major Science and Technology Special Projects in Hubei Province (2023BCA005), the Chief Scientist Research Project of Hubei Shizhen Laboratory (HSL2024SX0002), the postfunding project for major landmark achievements of "double first‐class" initiative at Beijing University of Chinese Medicine (BZXHBZ‐2025‐001), and the High‐Level Key Discipline of National Administration of Traditional Chinese Medicine—Traditional Chinese Constitutional Medicine (zyyzdxk‐2023251).

## Conflicts of Interest

The authors declare no conflicts of interest.

## Supporting information


**Supporting InformationSupplementary Materials** Additional supporting information can be found online in the Supporting Information section. Table S1: The TCM constitution classification and determination questionnaire (nine‐type scale with 60 items), including scoring rules and diagnostic thresholds. Table S2: Distribution characteristics of different mixed‐biased constitutions in the overall population and among those with/without new‐onset hyperglycemia. Table S3: Stepwise Cox proportional hazards regression models showing the association between TCM constitution and hyperglycemia with sequential adjustment for covariates. Table S4: Interaction terms between each TCM constitution type and age in the fully adjusted Cox model. Table S5: Association between TCM constitution and hyperglycemia risk stratified by age (18–39 and ≥ 40 years). Table S6: Association between TCM constitution and hyperglycemia risk stratified by follow‐up duration (< 24 and ≥ 24 months). Table S7: Association between TCM constitution and hyperglycemia risk stratified by follow‐up duration (6–30 and ≥ 30 months), excluding participants with < 6 months of follow‐up. Table S8: Sample sizes for each TCM constitution type before and after PSM (1:1, matched on age, sex, and BMI). Table S9: Association between TCM constitution and hyperglycemia risk after PSM. Table S10: Distribution characteristics of TCM constitution among participants with new‐onset prediabetes and diabetes. Table S11: Association between TCM constitution and risk of new‐onset prediabetes, excluding participants who progressed directly to diabetes.

## Data Availability

Research data are not shared.
