# Supplementary material for: TCM Constitution and the Risk of Hyperglycemia in Young and Middle‐Aged Adults: A Retrospective Cohort Study
Source: J Diabetes Res. 2026 Sep 23;2026:5548887. doi: 10.1155/jdr/5548887 (PMC13598545; doi:10.1155/jdr/5548887)
Supplement: Supplementary file 1 [file JDR-2026-5548887-s001.docx]

**Supplementary Table 1. The questionnaire of TCM constitution classification and determination. (Derived from Wang et al., 2025 ^[5]^(CC BY-NC 4.0), China Association of Chinese Medicine, 2009 ^[12]^)**.

Respondents must answer all items in the TCM Constitution Classification and Determination Scale (as shown in the table below). Each item is rated on a five-point scale. Raw scores and transformed scores are then calculated and used to determine constitutional type according to the criteria below.

Raw Score = Sum of all items scores.

Transformed Score = [(Raw Score – Number of Items in Each Cluster)/(Number of Items in Each Cluster × 4)] × 100.

If the transformed score of Balanced constitution ≥ 60 and all other eight types of constitution have transformed scores < 30, the individual is identified as Balanced constitution. If transformed score of each bias constitution ≥ 40, the corresponding bias constitution is identified. Coexistence of two or more types of constitution is allowed. According to the scoring rules, more symptoms and longer duration result in higher transformed scores.

### **Balanced Constitution (Type A)**

| **Please answer the following questions based on your experience and feelings over the past year.** | **Never**  **(Not at all)** | **Rarely**  **(A little)** | **Sometimes (Somewhat)** | **Frequently (Quite)** | **Always (Very)** |
| --- | --- | --- | --- | --- | --- |
| 1. Are you energetic? | 1 | 2 | 3 | 4 | 5 |
| 2. Do you get tired easily? * | 1 | 2 | 3 | 4 | 5 |
| 3. Do you speak in a low and weak voice? * | 1 | 2 | 3 | 4 | 5 |
| 4. Do you feel low-spirited or depressed? * | 1 | 2 | 3 | 4 | 5 |
| 5. Are you less tolerant of cold than most people (e.g., the cold of winter, the cool air of air conditioning in summer, electric fans, etc.)?* | 1 | 2 | 3 | 4 | 5 |
| 6. Can you adapt to changes in the natural and social environment? | 1 | 2 | 3 | 4 | 5 |
| 7. Do you have trouble sleeping? * | 1 | 2 | 3 | 4 | 5 |
| 8. Do you have trouble remembering things? * | 1 | 2 | 3 | 4 | 5 |

Result: □ Yes □ Mostly □ No

(Note: Items marked with * require reverse scoring: 1→5, 2→4, 3→3, 4→2, 5→1, before applying the transformation formula.)

### **Qi-Deficient Constitution (Type B)**

| **Please answer the following questions based on your experience and feelings over the past year.** | **Never**  **(Not at all)** | **Rarely**  **(A little)** | **Sometimes**  **(Somewhat)** | **Frequently (Quite)** | **Always (Very)** |
| --- | --- | --- | --- | --- | --- |
| 1. Do you get tired easily? | 1 | 2 | 3 | 4 | 5 |
| 2. Do you experience shortness of breath (difficulty breathing, feeling out of breath)? | 1 | 2 | 3 | 4 | 5 |
| 3. Do you experience palpitations? | 1 | 2 | 3 | 4 | 5 |
| 4. Do you experience dizziness or lightheadedness when standing up? | 1 | 2 | 3 | 4 | 5 |
| 5. Do you catch colds more easily than others? | 1 | 2 | 3 | 4 | 5 |
| 6. Do you prefer quiet or dislike talking? | 1 | 2 | 3 | 4 | 5 |
| 7. Do you speak in a low and weak voice? | 1 | 2 | 3 | 4 | 5 |
| 8. Do you tend to sweat spontaneously with only mild physical exertion? | 1 | 2 | 3 | 4 | 5 |

Result: □ Yes □ Tendency □ No

### **Yang-Deficient Constitution (Type C)**

| **Please answer the following questions based on your experience and feelings over the past year.** | **Never**  **(Not at all)** | **Rarely**  **(A little)** | **Sometimes**  **(Somewhat)** | **Frequently (Quite)** | **Always (Very)** |
| --- | --- | --- | --- | --- | --- |
| 1. Do you have cold hands and feet? | 1 | 2 | 3 | 4 | 5 |
| 2. Do you feel cold in your epigastrium, back, lower back, or knees? | 1 | 2 | 3 | 4 | 5 |
| 3. Do you feel chilly or wear more clothes than others? | 1 | 2 | 3 | 4 | 5 |
| 4. Are you less tolerant of cold than most people (e.g., the cold of winter, the cool air of air conditioning in summer, electric fans, etc.)? | 1 | 2 | 3 | 4 | 5 |
| 5. Do you catch colds more easily than others? | 1 | 2 | 3 | 4 | 5 |
| 6. Do you feel uncomfortable eating or drinking cold things, or tent to avoid them? | 1 | 2 | 3 | 4 | 5 |
| 7. Do you tend to get diarrhea (loose stools) after exposure to cold or after eating or drinking cold things? | 1 | 2 | 3 | 4 | 5 |

Result: □ Yes □ Tendency □ No

### **Yin-Deficient Constitution (Type D)**

| **Please answer the following questions based on your experience and feelings over the past year.** | **Never**  **(Not at all)** | **Rarely**  **(A little)** | **Sometimes**  **(Somewhat)** | **Frequently (Quite)** | **Always (Very)** |
| --- | --- | --- | --- | --- | --- |
| 1. Do you feel heat in your palms and soles? | 1 | 2 | 3 | 4 | 5 |
| 2. Do you feel heat in your body or face? | 1 | 2 | 3 | 4 | 5 |
| 3. Do you have dry skin or lips? | 1 | 2 | 3 | 4 | 5 |
| 4. Are your lips redder than most people’s? | 1 | 2 | 3 | 4 | 5 |
| 5. Are you prone to constipation or dry stools? | 1 | 2 | 3 | 4 | 5 |
| 6. Do you have red cheeks or a tendency to redness on your cheeks? | 1 | 2 | 3 | 4 | 5 |
| 7. Do you have dry eyes? | 1 | 2 | 3 | 4 | 5 |
| 8. Do you have a dry mouth and throat, or do you often feel thirsty? | 1 | 2 | 3 | 4 | 5 |

Result: □ Yes □ Tendency □ No

### **Phlegm-Dampness Constitution (Type E)**

| **Please answer the following questions based on your experience and feelings over the past year.** | **Never**  **(Not at all)** | **Rarely**  **(A little)** | **Sometimes**  **(Somewhat)** | **Frequently (Quite)** | **Always (Very)** |
| --- | --- | --- | --- | --- | --- |
| 1. Do you feel chest oppression or abdominal bloating? | 1 | 2 | 3 | 4 | 5 |
| 2. Do you feel a sense of heaviness or sluggishness in your body? | 1 | 2 | 3 | 4 | 5 |
| 3. Is your abdomen soft and distended? | 1 | 2 | 3 | 4 | 5 |
| 4. Does your forehead tend to look oily or shiny? | 1 | 2 | 3 | 4 | 5 |
| 5. Are your upper eyelids swollen compared to others (with a slight puffiness)? | 1 | 2 | 3 | 4 | 5 |
| 6. Do you have a sticky sensation in your mouth? | 1 | 2 | 3 | 4 | 5 |
| 7. Do you usually have excessive phlegm, especially a constant feeling of phlegm stuck in your throat? | 1 | 2 | 3 | 4 | 5 |
| 8. Do you have a thick, greasy tongue coating or a feeling of a thick coating? | 1 | 2 | 3 | 4 | 5 |

Result: □ Yes □ Tendency □ No

### **Damp-Heat Constitution (Type F)**

| **Please answer the following questions based on your experience and feelings over the past year.** | **Never**  **(Not at all)** | **Rarely**  **(A little)** | **Sometimes**  **(Somewhat)** | **Frequently (Quite)** | **Always (Very)** |
| --- | --- | --- | --- | --- | --- |
| 1. Do you have an oily sensation or shiny appearance on your face or nose? | 1 | 2 | 3 | 4 | 5 |
| 2. Do you tend to develop acne or boils? | 1 | 2 | 3 | 4 | 5 |
| 3. Do you notice a bitter taste in your mouth or an unpleasant taste or odor? | 1 | 2 | 3 | 4 | 5 |
| 4. Do you have sticky stools that are difficult to pass, with a feeling of incomplete evacuation? | 1 | 2 | 3 | 4 | 5 |
| 5. Do you notice a burning sensation when urinating, or find that your urine is dark or concentrated? | 1 | 2 | 3 | 4 | 5 |
| 6. Do you have yellow vaginal discharge? (Females only) | 1 | 2 | 3 | 4 | 5 |
| 7. Do you experience scrotal dampness or moisture? (Males only) | 1 | 2 | 3 | 4 | 5 |

Result: □ Yes □ Tendency □ No

### **Blood Stasis Constitution (Type G)**

| **Please answer the following questions based on your experience and feelings over the past year.** | **Never**  **(Not at all)** | **Rarely**  **(A little)** | **Sometimes**  **(Somewhat)** | **Frequently (Quite)** | **Always (Very)** |
| --- | --- | --- | --- | --- | --- |
| 1. Do you notice bruises appearing on your skin without apparent cause? | 1 | 2 | 3 | 4 | 5 |
| 2. Do you have fine red threads on both cheekbones? | 1 | 2 | 3 | 4 | 5 |
| 3. Do you have pain anywhere in your body? | 1 | 2 | 3 | 4 | 5 |
| 4. Is your complexion dull, or are you prone to brown spots? | 1 | 2 | 3 | 4 | 5 |
| 5. Do you often have dark circles under your eyes? | 1 | 2 | 3 | 4 | 5 |
| 6. Do you have trouble remembering things? | 1 | 2 | 3 | 4 | 5 |
| 7. Are your lips dusky in color? | 1 | 2 | 3 | 4 | 5 |

Result: □ Yes □ Tendency □ No

### **Qi Stagnation Constitution (Type H)**

| **Please answer the following questions based on your experience and feelings over the past year.** | **Never**  **(Not at all)** | **Rarely**  **(A little)** | **Sometimes**  **(Somewhat)** | **Frequently (Quite)** | **Always (Very)** |
| --- | --- | --- | --- | --- | --- |
| 1. Do you feel low-spirited or depressed? | 1 | 2 | 3 | 4 | 5 |
| 2. Do you easily feel nervous or anxious? | 1 | 2 | 3 | 4 | 5 |
| 3. Are you sentimental or emotionally vulnerable? | 1 | 2 | 3 | 4 | 5 |
| 4. Do you easily feel frightened or startled? | 1 | 2 | 3 | 4 | 5 |
| 5. Do you experience a feeling of fullness or distending pain in your sides or chest? | 1 | 2 | 3 | 4 | 5 |
| 6. Do you sigh for no reason? | 1 | 2 | 3 | 4 | 5 |
| 7. Do you have a sensation of a foreign body trapped in your throat that you can neither cough up nor swallow down? | 1 | 2 | 3 | 4 | 5 |

Result: □ Yes □ Tendency □ No

### **Intrinsic/Allergic Constitution (Type I)**

| **Please answer the following questions based on your experiences and feelings over the past year.** | **Never**  **(Not at all)** | **Rarely**  **(A little)** | **Sometimes**  **(Somewhat)** | **Frequently (Quite)** | **Always (Very)** |
| --- | --- | --- | --- | --- | --- |
| 1. Do you sneeze even when you do not have a cold? | 1 | 2 | 3 | 4 | 5 |
| 2. Do you have nasal congestion or a runny nose even when you do not have a cold? | 1 | 2 | 3 | 4 | 5 |
| 3. Do you wheeze or cough in response to seasonal changes, temperature shifts, or strong odors? | 1 | 2 | 3 | 4 | 5 |
| 4. Are you prone to allergies (to medication, food, smells, pollen, or during seasonal transitions/weather changes)? | 1 | 2 | 3 | 4 | 5 |
| 5. Is your skin prone to hives or wheals (urticaria)? | 1 | 2 | 3 | 4 | 5 |
| 6. Has your skin ever developed purplish-red spots or bruising (purpura) as an allergic reaction? | 1 | 2 | 3 | 4 | 5 |
| 7. Does your skin redden easily when scratched, leaving visible scratch marks? | 1 | 2 | 3 | 4 | 5 |

Result: □ Yes □ Tendency □ No

**Supplementary Table 2. Distribution characteristics of different mixed biased constitution.**

| **Mixed biased constitution** | **Overall** | **Newly onset hyperglycemia population (FBG≥5.6 mmol/L or HbA1c≥5.7%)** | |
| --- | --- | --- | --- |
|  |  | **No** | **Yes** |
| **Yang-deficient mixed (n, %)** | **n = 126** | **n = 91** | **n = 35** |
| Yang-deficient_Qi-deficient | 13(10.32) | 9(9.89) | 4(11.43) |
| Yang-deficient_Qi stagnation | 3(2.38) | 2(2.20) | 1(2.86) |
| Yang-deficient_Phlegm-dampness | 84(66.67) | 65(71.43) | 19(54.29) |
| Yang-deficient_Damp-heat | 14(11.11) | 9(9.89) | 5(14.29) |
| Yang-deficient_Blood stasis | 12(9.52) | 6(6.59) | 6(17.14) |
| Yang-deficient_Yin-deficient | 0(0.00) | 0(0.00) | 0(0.00) |
| **Qi-deficient mixed (n, %)** | **n = 737** | **n = 535** | **n = 202** |
| Yang-deficient_Qi-deficient | 13(1.76) | 9(1.68) | 4(1.98) |
| Qi-deficient_Qi stagnation | 20(2.71) | 14(2.62) | 6(2.97) |
| Qi-deficient_Phlegm-dampness | 477(64.72) | 347(64.86) | 130(64.36) |
| Qi-deficient_Damp-heat | 86(11.67) | 61(11.40) | 25(12.38) |
| Qi-deficient_Blood stasis | 42(5.70) | 33(6.17) | 9(4.46) |
| Qi-deficient_Yin-deficient | 99(13.43) | 71(13.27) | 28(13.86) |
| **Qi stagnation mixed (n, %)** | **n = 898** | **n = 588** | **n = 310** |
| Yang-deficient_Qi stagnation | 3(0.33) | 2(0.34) | 1(0.32) |
| Qi-deficient_Qi stagnation | 20(2.23) | 14(2.38) | 6(1.94) |
| Qi stagnation_Phlegm-dampness | 363(40.42) | 251(42.69) | 112(36.13) |
| Qi stagnation_Damp-heat | 225(25.06) | 139(23.64) | 86(27.74) |
| Qi stagnation_Blood stasis | 232(25.84) | 147(25.00) | 85(27.42) |
| Qi stagnation_Yin-deficient | 55(6.12) | 35(5.95) | 20(6.45) |
| **Phlegm-dampness mixed (n, %)** | **n = 1,621** | **n = 1,137** | **n = 484** |
| Yang-deficient_Phlegm-dampness | 84(5.18) | 65(5.72) | 19(3.93) |
| Qi-deficient_Phlegm-dampness | 477(29.43) | 347(30.52) | 130(26.86) |
| Qi stagnation_Phlegm-dampness | 363(22.39) | 251(22.08) | 112(23.14) |
| Phlegm-dampness_Damp-heat | 309(19.06) | 212(18.65) | 97(20.04) |
| Phlegm-dampness_Blood stasis | 379(23.38) | 256(22.52) | 123(25.41) |
| Phlegm-dampness_Yin-deficient | 9(0.56) | 6(0.53) | 3(0.62) |
| **Damp-heat mixed (n, %)** | **n = 1,410** | **n = 968** | **n = 442** |
| Yang-deficient_Damp-heat | 14(0.99) | 9(0.93) | 5(1.13) |
| Qi-deficient_Damp-heat | 86(6.10) | 61(6.30) | 25(5.66) |
| Qi stagnation_Damp-heat | 225(15.96) | 139(14.36) | 86(19.46) |
| Phlegm-dampness_Damp-heat | 309(21.91) | 212(21.90) | 97(21.95) |
| Damp-heat_Blood stasis | 717(50.85) | 504(52.07) | 213(48.19) |
| Damp-heat_Yin-deficient | 59(4.18) | 43(4.44) | 16(3.62) |
| **Blood stasis mixed (n, %)** | **n = 1,597** | **n = 1,098** | **n = 499** |
| Yang-deficient_Blood stasis | 12(0.75) | 6(0.55) | 6(1.20) |
| Qi-deficient_Blood stasis | 42(2.63) | 33(3.01) | 9(1.80) |
| Qi stagnation_Blood stasis | 232(14.53) | 147(13.39) | 85(17.03) |
| Phlegm-dampness_Blood stasis | 379(23.73) | 256(23.32) | 123(24.65) |
| Damp-heat_Blood stasis | 717(44.90) | 504(45.90) | 213(42.69) |
| Blood stasis_Yin-deficient | 215(13.46) | 152(13.84) | 63(12.63) |
| **Yin-deficient mixed (n, %)** | **n = 437** | **n = 307** | **n = 130** |
| Yang-deficient_Yin-deficient | 0(0.00) | 0(0.00) | 0(0.00) |
| Qi-deficient_Yin-deficient | 99(22.65) | 71(23.13) | 28(21.54) |
| Qi stagnation_Yin-deficient | 55(12.59) | 35(11.40) | 20(15.38) |
| Phlegm-dampness_Yin-deficient | 9(2.06) | 6(1.95) | 3(2.31) |
| Damp-heat_Yin-deficient | 59(13.50) | 43(14.01) | 16(12.31) |
| Blood stasis_Yin-deficient | 215(49.20) | 152(49.51) | 63(48.46) |

**Supplementary Table 3. Association between TCM constitution and hyperglycemia with sequential adjustment of covariates.**

| **Variables** | **Newly onset of hyperglycemia**  **sex**  **age**  **BMI**  **SBP**  **TC**  **TG**  **LDL**  **UA**  **SCr** | | | | | | | | | | | | | | | | | | | | | |
| --- | --- | --- | --- | --- | --- | --- | --- | --- | --- | --- | --- | --- | --- | --- | --- | --- | --- | --- | --- | --- | --- | --- |
|  | **HR (95%CI)** | | ***P* value** | **HR (95%CI)** | ***P* value** | **HR (95%CI)** | ***P* value** | | **HR (95%CI)** | ***P* value** | **HR (95%CI)** | ***P* value** | **HR (95%CI)** | ***P* value** | **HR (95%CI)** | ***P* value** | **HR (95%CI)** | ***P* value** | **HR (95%CI)** | ***P* value** | **HR (95%CI)** | ***P* value** |
| **Yang-deficient** | 2.66(1.61,4.40) | | <0.001 | 2.33(1.39,3.90) | 0.001 | 1.41(0.82,2.42) | 0.211 | | 1.40(0.76,2.57) | 0.278 | 1.49(0.80,2.80) | 0.212 | 1.63(0.86,3.10) | 0.136 | 1.65(0.87,3.12) | 0.127 | 2.05(1.02,4.09) | 0.043 | 2.24(1.12,4.48) | 0.023 | 2.27(1.13,4.57) | 0.021 |
| **Sex** |  | |  | 0.31(0.19,0.49) | <0.001 | 0.38(0.23,0.61) | <0.001 | | 0.42(0.24,0.73) | 0.002 | 0.40(0.22,0.71) | 0.002 | 0.43(0.25,0.77) | 0.004 | 0.45(0.25,0.81) | 0.007 | 0.82(0.42,1.60) | 0.562 | 0.55(0.25,1.24) | 0.152 | 0.49(0.18,1.35) | 0.168 |
| **Age** |  | |  |  |  | 1.06(1.04,1.08) | <0.001 | | 1.06(1.04,1.08) | <0.001 | 1.07(1.04,1.09) | <0.001 | 1.07(1.04,1.09) | <0.001 | 1.07(1.05,1.09) | <0.001 | 1.06(1.03,1.08) | <0.001 | 1.06(1.04,1.09) | <0.001 | 1.07(1.04,1.10) | <0.001 |
| **BMI** |  | |  |  |  |  |  | | 1.07(0.96,1.18) | 0.230 | 1.08(0.97,1.20) | 0.156 | 1.05(0.95,1.16) | 0.378 | 1.02(0.92,1.13) | 0.694 | 1.00(0.88,1.13) | 0.983 | 1.06(0.92,1.22) | 0.447 | 1.06(0.92,1.22) | 0.436 |
| **SBP** |  | |  |  |  |  |  | |  |  | 0.99(0.96,1.01) | 0.437 | 0.99(0.96,1.01) | 0.388 | 0.99(0.97,1.01) | 0.304 | 1.00(0.97,1.02) | 0.774 | 0.99(0.96,1.01) | 0.372 | 0.99(0.96,1.02) | 0.410 |
| **TC** |  | |  |  |  |  |  | |  |  |  |  | 1.40(1.07,1.83) | 0.015 | 1.32(1.00,1.74) | 0.052 | 0.76(0.39,1.49) | 0.426 | 0.73(0.37,1.44) | 0.366 | 0.73(0.38,1.44) | 0.365 |
| **TG** |  | |  |  |  |  |  | |  |  |  |  |  |  | 1.23(0.93,1.63) | 0.146 | 1.47(1.01,2.15) | 0.045 | 1.64(1.10,2.43) | 0.015 | 1.63(1.09,2.43) | 0.016 |
| **LDL** |  | |  |  |  |  |  | |  |  |  |  |  |  |  |  | 1.84(0.80,4.23) | 0.151 | 1.99(0.87,4.52) | 0.102 | 1.99(0.87,4.54) | 0.102 |
| **UA** |  | |  |  |  |  |  | |  |  |  |  |  |  |  |  |  |  | 1.00(0.99,1.00) | 0.101 | 1.00(0.99,1.00) | 0.134 |
| **SCr** |  | |  |  |  |  |  | |  |  |  |  |  |  |  |  |  |  |  |  | 0.99(0.96,1.03) | 0.699 |
| **Qi-deficient** | 1.95(1.39,2.73) | | <0.001 | 1.61(1.14,2.28) | 0.007 | 1.15(0.80,1.65) | 0.451 | | 1.02(0.69,1.52) | 0.922 | 1.02(0.67,1.53) | 0.943 | 1.02(0.67,1.54) | 0.928 | 1.02(0.67,1.54) | 0.927 | 1.00(0.64,1.55) | 0.983 | 0.99(0.64,1.54) | 0.967 | 0.98(0.63,1.53) | 0.928 |
| **Sex** |  | |  | 0.39(0.28,0.55) | <0.001 | 0.51(0.36,0.71) | <0.001 | | 0.54(0.36,0.79) | 0.002 | 0.49(0.32,0.75) | 0.001 | 0.53(0.35,0.80) | 0.003 | 0.53(0.34,0.81) | 0.003 | 0.83(0.53,1.31) | 0.434 | 1.00(0.58,1.70) | 0.988 | 0.76(0.39,1.51) | 0.439 |
| **Age** |  | |  |  |  | 1.05(1.04,1.07) | <0.001 | | 1.05(1.04,1.07) | <0.001 | 1.06(1.04,1.07) | <0.001 | 1.05(1.04,1.07) | <0.001 | 1.05(1.04,1.07) | <0.001 | 1.04(1.02,1.06) | <0.001 | 1.04(1.02,1.06) | <0.001 | 1.04(1.02,1.06) | <0.001 |
| **BMI** |  | |  |  |  |  |  | | 1.07(1.00,1.15) | 0.057 | 1.08(1.00,1.16) | 0.040 | 1.08(1.01,1.17) | 0.035 | 1.08(1.00,1.17) | 0.037 | 1.05(0.97,1.14) | 0.251 | 1.04(0.96,1.13) | 0.354 | 1.04(0.96,1.14) | 0.322 |
| **SBP** |  | |  |  |  |  |  | |  |  | 0.99(0.98,1.01) | 0.260 | 0.99(0.98,1.01) | 0.262 | 0.99(0.98,1.01) | 0.262 | 1.00(0.98,1.01) | 0.754 | 1.00(0.98,1.01) | 0.765 | 1.00(0.98,1.01) | 0.725 |
| **TC** |  | |  |  |  |  |  | |  |  |  |  | 1.16(0.95,1.42) | 0.135 | 1.16(0.95,1.43) | 0.151 | 0.74(0.43,1.26) | 0.265 | 0.76(0.44,1.30) | 0.313 | 0.76(0.44,1.31) | 0.318 |
| **TG** |  | |  |  |  |  |  | |  |  |  |  |  |  | 1.00(0.85,1.17) | 0.977 | 1.12(0.88,1.44) | 0.351 | 1.09(0.85,1.40) | 0.498 | 1.11(0.86,1.44) | 0.424 |
| **LDL** |  | |  |  |  |  |  | |  |  |  |  |  |  |  |  | 1.76(0.92,3.34) | 0.087 | 1.66(0.87,3.19) | 0.126 | 1.68(0.87,3.25) | 0.123 |
| **UA** |  | |  |  |  |  |  | |  |  |  |  |  |  |  |  |  |  | 1.00(1.00,1.00) | 0.222 | 1.00(1.00,1.00) | 0.174 |
| **SCr** |  | |  |  |  |  |  | |  |  |  |  |  |  |  |  |  |  |  |  | 0.99(0.97,1.01) | 0.220 |
| **Qi stagnation** | 3.52(2.41,5.14) | | <0.001 | 2.66(1.71,4.13) | <0.001 | 2.13(1.36,3.32) | 0.001 | | 2.15(1.31,3.53) | 0.002 | 2.25(1.34,3.75) | 0.002 | 2.26(1.35,3.78) | 0.002 | 2.27(1.36,3.79) | 0.002 | 1.91(1.13,3.25) | 0.016 | 1.91(1.13,3.24) | 0.016 | 1.91(1.13,3.24) | 0.016 |
| **Sex** |  | |  | 0.61(0.40,0.92) | 0.019 | 0.74(0.49,1.14) | 0.175 | | 0.65(0.39,1.07) | 0.087 | 0.63(0.37,1.06) | 0.082 | 0.66(0.39,1.11) | 0.115 | 0.65(0.39,1.10) | 0.112 | 0.88(0.59,1.53) | 0.659 | 0.89(0.48,1.66) | 0.715 | 0.76(0.36,1.62) | 0.481 |
| **Age** |  | |  |  |  | 1.06(1.04,1.08) | <0.001 | | 1.07(1.05,1.09) | <0.001 | 1.07(1.05,1.09) | <0.001 | 1.07(1.05,1.09) | <0.001 | 1.07(1.04,1.08) | <0.001 | 1.06(1.04,1.08) | <0.001 | 1.06(1.04,1.08) | <0.001 | 1.06(1.04,1.08) | <0.001 |
| **BMI** |  | |  |  |  |  |  | | 1.00(0.93,1.08) | 0.969 | 1.01(0.94,1.10) | 0.833 | 0.99(0.93,1.08) | 0.934 | 1.00(0.92,1.09) | 0.974 | 1.00(0.92,1.10) | 0.948 | 1.00(0.92,1.10) | 0.959 | 1.01(0.92,1.10) | 0.894 |
| **SBP** |  | |  |  |  |  |  | |  |  | 1.00(0.98,1.01) | 0.709 | 0.99(0.98,1.01) | 0.575 | 0.99(0.98,1.01) | 0.564 | 1.00(0.98,1.02) | 0.939 | 1.00(0.98,1.02) | 0.940 | 1.00(0.98,1.02) | 0.944 |
| **TC** |  | |  |  |  |  |  | |  |  |  |  | 1.18(0.96,1.45) | 0.107 | 1.22(0.97,1.53) | 0.087 | 1.03(0.61,1.76) | 0.905 | 1.03(0.60,1.77) | 0.902 | 1.05(0.61,1.78) | 0.869 |
| **TG** |  | |  |  |  |  |  | |  |  |  |  |  |  | 0.97(0.87,1.08) | 0.562 | 1.00(0.85,1.17) | 0.995 | 1.00(0.85,1.18) | 0.996 | 0.99(0.84,1.17) | 0.900 |
| **LDL** |  | |  |  |  |  |  | |  |  |  |  |  |  |  |  | 1.18(0.61,2.27) | 0.630 | 1.17(0.60,2.28) | 0.636 | 1.16(0.60,2.25) | 0.661 |
| **UA** |  | |  |  |  |  |  | |  |  |  |  |  |  |  |  |  |  | 1.00(0.99,1.00) | 0.963 | 1.00(1.00,1.00) | 0.792 |
| **SCr** |  | |  |  |  |  |  | |  |  |  |  |  |  |  |  |  |  |  |  | 0.99(0.97,1.01) | 0.473 |
| **Phlegm-dampness** | 3.13(2.30,4.25) | | <0.001 | 2.62(1.91,3.61) | <0.001 | 2.31(1.68,3.18) | <0.001 | | 2.08(1.43,3.02) | <0.001 | 2.18(1.48,3.20) | <0.001 | 2.20(1.49,3.24) | <0.001 | 2.21(1.50,3.25) | <0.001 | 1.65(1.09,2.49) | 0.017 | 1.63(1.08,2.47) | 0.019 | 1.65(1.10,2.49) | 0.018 |
| **Sex** |  | |  | 0.58(0.45,0.74) | <0.001 | 0.72(0.55,0.92) | 0.010 | | 0.69(0.52,0.90) | 0.008 | 0.75(0.56,0.99) | 0.045 | 0.78(0.59,1.04) | 0.090 | 0.79(0.59,1.05) | 0.106 | 1.14(0.84,1.55) | 0.403 | 1.18(0.82,1.68) | 0.376 | 0.99(0.62,1.57) | 0.973 |
| **Age** |  | |  |  |  | 1.05(1.04,1.06) | <0.001 | | 1.05(1.04,1.06) | <0.001 | 1.05(1.03,1.07) | <0.001 | 1.05(1.03,1.06) | <0.001 | 1.05(1.03,1.06) | <0.001 | 1.04(1.02,1.05) | <0.001 | 1.04(1.02,1.05) | <0.001 | 1.04(1.02,1.05) | <0.001 |
| **BMI** |  | |  |  |  |  |  | | 1.04(1.00,1.08) | 0.078 | 1.03(0.98,1.07) | 0.260 | 1.02(0.97,1.07) | 0.460 | 1.00(0.97,1.06) | 0.574 | 0.99(0.95,1.05) | 0.916 | 0.99(0.94,1.05) | 0.859 | 0.99(0.94,1.05) | 0.848 |
| **SBP** |  | |  |  |  |  |  | |  |  | 1.01(1.00,1.02) | 0.007 | 1.01(1.00,1.02) | 0.009 | 1.01(1.00,1.02) | 0.010 | 1.01(1.00,1.02) | 0.014 | 1.02(1.00,1.02) | 0.015 | 1.01(1.00,1.02) | 0.015 |
| **TC** |  | |  |  |  |  |  | |  |  |  |  | 1.18(1.06,1.33) | 0.003 | 1.16(1.03,1.31) | 0.016 | 0.66(0.46,0.94) | 0.021 | 0.67(0.47,0.96) | 0.027 | 0.66(0.46,0.95) | 0.025 |
| **TG** |  | |  |  |  |  |  | |  |  |  |  |  |  | 1.04(0.95,1.14) | 0.438 | 1.22(1.07,1.39) | 0.002 | 1.21(1.06,1.39) | 0.004 | 1.21(1.06,1.38) | 0.004 |
| **LDL** |  | |  |  |  |  |  | |  |  |  |  |  |  |  |  | 2.03(1.29,3.19) | 0.002 | 2.07(1.27,3.17) | 0.003 | 2.04(1.29,3.24) | 0.002 |
| **UA** |  | |  |  |  |  |  | |  |  |  |  |  |  |  |  |  |  | 1.00(0.99,1.00) | 0.741 | 1.00(0.99,1.02) | 0.671 |
| **SCr** |  | |  |  |  |  |  | |  |  |  |  |  |  |  |  |  |  |  |  | 0.99(0.98,1.01) | 0.256 |
| **Damp-heat** | 2.78(2.09,3.70) | | <0.001 | 2.25(1.68,3.02) | <0.001 | 1.98(1.48,2.65) | <0.001 | | 1.84(1.34,2.53) | <0.001 | 1.92(1.39,2.67) | <0.001 | 1.93(1.39,2.67) | <0.001 | 1.93(1.39,2.67) | <0.001 | 1.49(1.03,2.15) | 0.032 | 1.49(1.03,2.14) | 0.034 | 1.48(1.03,3.14) | 0.035 |
| **Sex** |  | |  | 0.52(0.45,0.61) | <0.001 | 0.62(0.53,0.73) | <0.001 | | 0.74(0.61,0.88) | 0.001 | 0.73(0.61,0.89) | 0.001 | 0.75(0.63,0.91) | 0.003 | 0.77(0.64,0.93) | 0.008 | 1.04(0.86,1.27) | 0.675 | 1.07(0.85,1.34) | 0.583 | 0.99(0.75,1.31) | 0.938 |
| **Age** |  | |  |  |  | 1.04(1.03,1.05) | <0.001 | | 1.04(1.03,1.04) | <0.001 | 1.04(1.03,1.04) | <0.001 | 1.03(1.02,1.04) | <0.001 | 1.03(1.02,1.04) | <0.001 | 1.03(1.02,1.04) | <0.001 | 1.03(1.02,1.04) | <0.001 | 1.03(1.02,1.04) | <0.001 |
| **BMI** |  | |  |  |  |  |  | | 1.09(1.06,1.12) | <0.001 | 1.09(1.06,1.12) | <0.001 | 1.09(1.06,1.12) | <0.001 | 1.08(1.05,1.13) | <0.001 | 1.06(1.03,1.09) | <0.001 | 1.06(1.03,1.09) | <0.001 | 1.07(1.03,1.09) | <0.001 |
| **SBP** |  | |  |  |  |  |  | |  |  | 1.00(0.99,1.01) | 0.566 | 1.00(0.99,1.01) | 0.736 | 1.00(0.99,1.01) | 0.765 | 1.00(0.99,1.01) | 0.265 | 1.00(0.99,1.01) | 0.274 | 1.00(0.99,1.02) | 0.302 |
| **TC** |  | |  |  |  |  |  | |  |  |  |  | 1.17(1.08,1.27) | <0.001 | 1.16(1.07,1.25) | <0.001 | 0.69(0.54,0.90) | 0.005 | 0.70(0.53,0.90) | 0.005 | 0.70(0.54,0.90) | 0.006 |
| **TG** |  | |  |  |  |  |  | |  |  |  |  |  |  | 1.05(0.98,1.13) | 0.144 | 1.10(1.02,1.19) | 0.019 | 1.06(1.01,1.19) | 0.024 | 1.09(1.01,1.18) | 0.035 |
| **LDL** |  | |  |  |  |  |  | |  |  |  |  |  |  |  |  | 1.93(1.40,2.67) | <0.001 | 1.92(1.39,2.66) | <0.001 | 1.92(1.39,2.65) | <0.001 |
| **UA** |  | |  |  |  |  |  | |  |  |  |  |  |  |  |  |  |  | 1.00(1.00,1.00) | 0.711 | 1.00(1.00,1.00) | 0.571 |
| **SCr** |  | |  |  |  |  |  | |  |  |  |  |  |  |  |  |  |  |  |  | 0.97(0.99,1.00) | 0.359 |
| **Blood stasis** | 4.25(2.92,6.20) | | <0.001 | 2.70(1.76,4.12) | <0.001 | 2.10(1.38,3.19) | 0.001 | | 2.08(1.33,3.26) | 0.001 | 2.05(1.28,3.29) | 0.003 | 2.06(1.29,3.29) | 0.003 | 2.06(1.28,3.29) | 0.003 | 2.23(1.35,3.69) | 0.002 | 2.25(1.36,3.73) | 0.002 | 2.30(1.39,3.82) | 0.001 |
| **Sex** |  | |  | 0.38(0.25,0.57) | <0.001 | 0.41(0.28,0.62) | <0.001 | | 0.41(0.26,0.64) | <0.001 | 0.36(0.23,0.59) | <0.001 | 0.38(0.24,0.61) | <0.001 | 0.39(0.24,0.63) | <0.001 | 0.60(0.36,1.00) | 0.052 | 0.52(0.29,0.93) | 0.028 | 0.43(0.21,0.89) | 0.022 |
| **Age** |  | |  |  |  | 1.06(1.04,1.07) | <0.001 | | 1.06(1.04,1.08) | <0.001 | 1.06(1.04,1.08) | <0.001 | 1.06(1.04,1.08) | <0.001 | 1.06(1.04,1.08) | <0.001 | 1.05(1.03,1.07) | <0.001 | 1.05(1.03,1.07) | <0.001 | 1.05(1.03,1.08) | <0.001 |
| **BMI** |  | |  |  |  |  |  | | 1.03(0.96,1.11) | 0.339 | 1.04(0.97,1.12) | 0.237 | 1.04(0.97,1.12) | 0.292 | 1.03(0.96,1.11) | 0.417 | 1.06(0.95,1.13) | 0.398 | 1.04(0.96,1.14) | 0.347 | 1.04(0.96,1.20) | 0.349 |
| **SBP** |  | |  |  |  |  |  | |  |  | 0.99(0.98,1.01) | 0.377 | 0.99(0.98,1.01) | 0.285 | 0.99(0.98,1.01) | 0.215 | 0.99(0.98,1.01) | 0.400 | 0.99(0.98,1.01) | 0.367 | 0.99(0.98,1.01) | 0.316 |
| **TC** |  | |  |  |  |  |  | |  |  |  |  | 1.17(0.97,1.40) | 0.099 | 1.13(0.94,1.37) | 0.203 | 0.88(0.54,1.46) | 0.629 | 0.86(0.52,1.47) | 0.560 | 0.88(0.53,1.50) | 0.616 |
| **TG** |  | |  |  |  |  |  | |  |  |  |  |  |  | 1.12(0.94,1.33) | 0.205 | 1.14(0.94,1.39) | 0.178 | 1.17(0.96,1.43) | 0.119 | 1.15(0.94,2.43) | 0.163 |
| **LDL** |  | |  |  |  |  |  | |  |  |  |  |  |  |  |  | 1.38(0.72,2.63) | 0.330 | 1.47(0.76,2.85) | 0.254 | 1.46(0.75,2.84) | 0.261 |
| **UA** |  | |  |  |  |  |  | |  |  |  |  |  |  |  |  |  |  | 0.99(0.99,1.00) | 0.319 | 0.99(0.99,1.00) | 0.380 |
| **SCr** |  | |  |  |  |  |  | |  |  |  |  |  |  |  |  |  |  |  |  | 0.99(0.98,1.01) | 0.385 |
| **Yin-deficient** | 1.94(1.33,2.83) | | 0.001 | 1.97(1.35,2.87) | <0.001 | 1.26(0.85,1.86) | | 0.244 | 1.26(0.83,1.92) | 0.275 | 1.24(0.80,1.93) | 0.339 | 1.19(0.76,1.88) | 0.438 | 1.18(0.75,1.87) | 0.458 | 0.99(0.59,1.66) | 0.975 | 0.93(0.55,1.56) | 0.777 | 0.92(0.55,1.56) | 0.783 |
| **sex** |  | |  | 0.51(0.34,0.78) | 0.002 | 0.52(0.34,0.80) | | 0.003 | 0.56(0.36,0.88) | 0.013 | 0.59(0.36,0.97) | 0.037 | 0.60(0.37,0.99) | 0.045 | 0.63(0.38,1.05) | 0.077 | 0.96(0.54,1.72) | 0.891 | 1.44(0.72,2.91) | 0.304 | 1.27(0.55,2.93) | 0.570 |
| **Age** |  | |  |  |  | 1.07(1.06,1.09) | | <0.001 | 1.07(1.05,1.09) | <0.001 | 1.08(1.05,110) | <0.001 | 1.07(1.05,1.10) | <0.001 | 1.07(1.05,1.09) | <0.001 | 1.06(1.04,1.09) | <0.001 | 1.06(1.04,1.09) | <0.001 | 1.06(1.04,1.09) | <0.001 |
| **BMI** |  | |  |  |  |  | |  | 1.06(0.99,1.15) | 0.094 | 1.08(1.00,1.17) | 0.046 | 1.07(0.99,1.16) | 0.075 | 1.07(0.99,1.16) | 0.099 | 1.04(0.95,1.13) | 0.411 | 1.01(0.92,1.11) | 0.880 | 1.01(0.92,1.10) | 0.883 |
| **SBP** |  | |  |  |  |  | |  |  |  | 0.99(0.98,1.01) | 0.501 | 0.99(0.98,1.01) | 0.510 | 0.99(0.98,1.01) | 0.483 | 1.00(0.99,1.02) | 0.742 | 1.01(0.99,1.02) | 0.513 | 1.01(0.99,1.23) | 0.521 |
| **TC** |  | |  |  |  |  |  | |  |  |  |  | 1.30(1.05,1.61) | 0.018 | 1.27(1.01,1.59) | 0.040 | 0.86(0.45,1.63) | 0.636 | 0.93(0.49,1.79) | 0.839 | 0.91(0.47,1.77) | 0.792 |
| **TG** |  | |  |  |  |  |  | |  |  |  |  |  |  | 1.12(0.83,1.51) | 0.466 | 1.22(0.85,1.76) | 0.284 | 1.08(0.72,1.60) | 0.735 | 1.07(0.72,1.60) | 0.726 |
| **LDL** |  | |  |  |  |  |  | |  |  |  |  |  |  |  |  | 1.55(0.70,3.41) | 0.278 | 1.38(0.62,3.10) | 0.431 | 1.41(0.62,3.19) | 0.414 |
| **UA** |  | |  |  |  |  |  | |  |  |  |  |  |  |  |  |  |  | 1.00(1.00,1.01) | 0.030 | 1.00(1.00,1.01) | 0.026 |
| **SCr** |  | |  |  |  |  |  | |  |  |  |  |  |  |  |  |  |  |  |  | 0.99(0.97,1.02) | 0.584 |
| **Yang-deficient mixed** | 2.84(1.82,4.43) | | <0.001 | 2.17(1.35,3.49) | 0.001 | 1.79(1.10,2.91) | 0.019 | | 1.61(0.92,2.82) | 0.097 | 1.67(0.94,2.97) | 0.081 | 1.68(0.96,2.95) | 0.071 | 1.72(0.98,3.02) | 0.059 | 1.78(0.96,3.32) | 0.065 | 1.80(0.97,3.35) | 0.063 | 1.87(1.00,3.49) | 0.050 |
| **Sex** |  | |  | 0.45(0.29,0.73) | 0.001 | 0.63(0.39,1.03) | 0.067 | | 0.61(0.34,1.10) | 0.102 | 0.53(0.29,0.99) | 0.046 | 0.61(0.33,1.12) | 0.112 | 0.68(0.36,1.26) | 0.220 | 1.26(0.64,2.49) | 0.502 | 0.98(0.41,2.32) | 0.958 | 1.41(0.52,3.85) | 0.506 |
| **Age** |  | |  |  |  | 1.07(1.05,1.09) | <0.001 | | 1.07(1.04,1.09) | <0.001 | 1.07(1.05,1.09) | <0.001 | 1.07(1.05,1.10) | <0.001 | 1.07(1.05,1.10) | <0.001 | 1.07(1.03,1.09) | <0.001 | 1.06(1.03,1.09) | <0.001 | 1.06(1.03,1.09) | <0.001 |
| **BMI** |  | |  |  |  |  |  | | 1.04(0.94,1.15) | 0.430 | 1.06(0.96,1.17) | 0.248 | 1.08(0.97,1.19) | 0.146 | 1.06(0.95,1.16) | 0.333 | 1.01(0.89,1.14) | 0.926 | 1.02(0.90,1.16) | 0.735 | 1.02(0.89,1.16) | 0.803 |
| **SBP** |  | |  |  |  |  |  | |  |  | 0.98(0.96,1.00) | 0.064 | 0.98(0.96,1.00) | 0.039 | 0.98(0.96,1.00) | 0.032 | 0.99(0.97,1.01) | 0.264 | 0.99(0.96,1.01) | 0.196 | 0.99(0.96,1.01) | 0.189 |
| **TC** |  | |  |  |  |  |  | |  |  |  |  | 1.38(1.09,1.75) | 0.008 | 1.34(1.06,1.69) | 0.015 | 0.99(0.50,1.98) | 0.981 | 0.96(0.48,1.93) | 0.911 | 0.93(0.46,1.82) | 0.848 |
| **TG** |  | |  |  |  |  |  | |  |  |  |  |  |  | 1.33(1.03,1.71) | 0.030 | 1.61(1.14,2.28) | 0.007 | 1.68(1.17,2.40) | 0.005 | 1.67(1.17,2.38) | 0.005 |
| **LDL** |  | |  |  |  |  |  | |  |  |  |  |  |  |  |  | 1.53(0.66,3.53) | 0.318 | 1.59(0.68,3.68) | 0.282 | 1.63(0.71,3.76) | 0.253 |
| **UA** |  | |  |  |  |  |  | |  |  |  |  |  |  |  |  |  |  | 1.00(0.99,1.00) | 0.350 | 0.99(0.99,1.00) | 0.233 |
| **SCr** |  | |  |  |  |  |  | |  |  |  |  |  |  |  |  |  |  |  |  | 1.02(0.99,1.05) | 0.192 |
| **Qi-deficient mixed** | 2.66(1.95,3.64) | | <0.001 | 2.18(1.57,3.04) | <0.001 | 1.79(1.29,2.50) | 0.001 | | 1.59(1.10,2.29) | 0.013 | 1.63(1.11,2.37) | 0.012 | 1.64(1.12,2.40) | 0.011 | 1.64(1.12,2.40) | 0.011 | 1.28(0.85,1.93) | 0.230 | 1.25(0.83,1.88) | 0.290 | 1.25(0.83,1.88) | 0.290 |
| **Sex** |  | |  | 0.61(0.47,0.81) | <0.001 | 0.65(0.50,0.86) | 0.002 | | 0.71(0.51,0.98) | 0.037 | 0.70(0.50,0.98) | 0.040 | 0.75(0.53,1.05) | 0.095 | 0.75(0.53,1.05) | 0.094 | 1.01(0.70,1.45) | 0.973 | 1.21(0.80,1.85) | 0.362 | 1.17(0.68,2.01) | 0.561 |
| **Age** |  | |  |  |  | 1.05(1.04,1.07) | <0.001 | | 1.05(1.04,1.07) | <0.001 | 1.05(1.04,1.07) | <0.001 | 1.05(1.03,1.07) | <0.001 | 1.05(1.03,1.07) | <0.001 | 1.04(1.03,1.06) | <0.001 | 1.04(1.03,1.06) | <0.001 | 1.04(1.03,1.06) | <0.001 |
| **BMI** |  | |  |  |  |  |  | | 1.07(1.02,1.12) | 0.004 | 1.07(1.02,1.12) | 0.003 | 1.07(1.02,1.12) | 0.004 | 1.07(1.02,1.12) | 0.004 | 1.07(1.01,1.12) | 0.010 | 1.05(1.00,1.11) | 0.043 | 1.05(1.00,1.11) | 0.045 |
| **SBP** |  | |  |  |  |  |  | |  |  | 1.00(0.99,1.01) | 0.985 | 1.00(0.99,1.01) | 0.896 | 1.00(0.99,1.01) | 0.896 | 1.00(0.99,1.01) | 0.723 | 1.00(0.99,1.02) | 0.615 | 1.00(0.99,1.02) | 0.616 |
| **TC** |  | |  |  |  |  |  | |  |  |  |  | 1.21(1.05,1.40) | 0.009 | 1.21(1.04,1.42) | 0.016 | 0.88(0.58,1.35) | 0.563 | 0.92(0.60,1.41) | 0.716 | 0.93(0.61,1.42) | 0.730 |
| **TG** |  | |  |  |  |  |  | |  |  |  |  |  |  | 1.00(0.93,1.07) | 0.961 | 1.04(0.95,1.13) | 0.398 | 1.03(0.94,1.12) | 0.533 | 1.03(0.94,1.12) | 0.541 |
| **LDL** |  |  |  |  |  |  |  | |  |  |  |  |  |  |  |  | 1.49(0.88,2.54) | 0.141 | 1.36(0.80,2.34) | 0.259 | 1.36(0.79,2.33) | 0.263 |
| **UA** |  |  |  |  |  |  |  | |  |  |  |  |  |  |  |  |  |  | 1.00(1.00,1.00) | 0.074 | 1.00(1.00,1.00) | 0.073 |
| **SCr** |  |  |  |  |  |  |  | |  |  |  |  |  |  |  |  |  |  |  |  | 1.00(0.98,1.01) | 0.840 |
| **Qi stagnation mixed** | 4.12(3.02,5.61) | | <0.001 | 3.14(2.25,4.38) | <0.001 | 2.45(1.75,3.43) | <0.001 | | 2.20(1.52,3.18) | <0.001 | 2.31(1.58,3.39) | <0.001 | 2.36(1.61,3.47) | <0.001 | 2.36(1.61,3.47) | <0.001 | 1.90(1.26,2.88) | 0.002 | 1.88(1.24,2.84) | 0.003 | 1.87(1.24,2.83) | 0.003 |
| **Sex** |  | |  | 0.57(0.45,0.74) | <0.001 | 0.60(0.47,0.77) | <0.001 | | 0.63(0.48,0.84) | 0.001 | 0.63(0.47,0.84) | 0.002 | 0.67(0.50,0.89) | 0.006 | 0.68(0.51,0.91) | 0.009 | 0.73(0.53,0.99) | 0.043 | 0.79(0.57,1.11) | 0.175 | 0.71(0.45,1.11) | 0.137 |
| **Age** |  | |  |  |  | 1.04(1.03,1.06) | <0.001 | | 1.04(1.03,1.05) | <0.001 | 1.04(1.03,1.05) | <0.001 | 1.04(1.02,1.05) | <0.001 | 1.04(1.02,1.05) | <0.001 | 1.03(1.02,1.05) | <0.001 | 1.03(1.02,1.05) | <0.001 | 1.03(1.02,1.05) | <0.001 |
| **BMI** |  | |  |  |  |  |  | | 1.08(1.04,1.12) | <0.001 | 1.08(1.04,1.13) | <0.001 | 1.08(1.04,1.12) | <0.001 | 1.07(1.03,1.12) | 0.001 | 1.08(1.04,1.13) | <0.001 | 1.07(1.03,1.12) | 0.001 | 1.07(1.03,1.12) | 0.001 |
| **SBP** |  | |  |  |  |  |  | |  |  | 1.00(0.99,1.01) | 0.997 | 1.00(0.99,1.01) | 0.702 | 1.00(0.99,1.01) | 0.653 | 1.00(0.99,1.01) | 0.793 | 1.00(0.99,1.01) | 0.830 | 1.00(0.99,1.01) | 0.845 |
| **TC** |  | |  |  |  |  |  | |  |  |  |  | 1.24(1.10,1.40) | <0.001 | 1.23(1.08,1.38) | 0.001 | 1.47(1.03,2.09) | 0.035 | 1.50(1.05,2.15) | 0.027 | 1.51(1.05,2.16) | 0.025 |
| **TG** |  | |  |  |  |  |  | |  |  |  |  |  |  | 1.06(0.94,1.20) | 0.350 | 1.04(0.92,1.18) | 0.521 | 1.03(0.90,1.17) | 0.679 | 1.02(0.90,1.17) | 0.746 |
| **LDL** |  | |  |  |  |  |  | |  |  |  |  |  |  |  |  | 0.76(0.48,1.21) | 0.251 | 0.74(0.47,1.18) | 0.211 | 0.74(0.47,1.18) | 0.211 |
| **UA** |  | |  |  |  |  |  | |  |  |  |  |  |  |  |  |  |  | 1.00(1.00,1.00) | 0.195 | 1.00(1.00,1.00) | 0.167 |
| **SCr** |  | |  |  |  |  |  | |  |  |  |  |  |  |  |  |  |  |  |  | 1.00(0.98,1.01) | 0.473 |
| **Phlegm-dampness mixed** | 3.28(2.45,4.41) | | <0.001 | 2.61(1.91,3.59) | <0.001 | 2.20(1.60,3.01) | <0.001 | | 2.01(1.42,2.84) | <0.001 | 2.06(1.44,2.94) | <0.001 | 2.08(1.46,2.97) | <0.001 | 2.08(1.46,2.97) | <0.001 | 1.45(0.99,2.13) | 0.055 | 1.42(0.97,2.08) | 0.073 | 1.42(0.97,2.08) | 0.073 |
| **Sex** |  | |  | 0.66(0.54,0.82) | <0.001 | 0.72(0.58,0.89) | 0.002 | | 0.78(0.61,0.99) | 0.045 | 0.75(0.58,0.96) | 0.023 | 0.77(0.60,0.99) | 0.041 | 0.77(0.60,0.99) | 0.041 | 0.93(0.72,1.20) | 0.555 | 1.11(0.83,1.49) | 0.462 | 1.11(0.77,1.60) | 0.573 |
| **Age** |  | |  |  |  | 1.05(1.04,1.06) | <0.001 | | 1.05(1.04,1.06) | <0.001 | 1.05(1.04,1.06) | <0.001 | 1.05(1.04,1.06) | <0.001 | 1.05(1.04,1.06) | <0.001 | 1.04(1.03,1.06) | <0.001 | 1.05(1.04,1.06) | <0.001 | 1.05(1.04,1.06) | <0.001 |
| **BMI** |  | |  |  |  |  |  | | 1.06(1.03,1.10) | <0.001 | 1.07(1.04,1.11) | <0.001 | 1.06(1.03,1.10) | <0.001 | 1.06(1.03,1.10) | <0.001 | 1.06(1.03,1.10) | <0.001 | 1.05(1.01,1.09) | 0.007 | 1.05(1.01,1.09) | 0.007 |
| **SBP** |  | |  |  |  |  |  | |  |  | 0.99(0.99,1.00) | 0.134 | 0.99(0.99,1.00) | 0.093 | 0.99(0.99,1.00) | 0.095 | 0.99(0.99,1.00) | 0.163 | 0.99(0.99,1.00) | 0.186 | 0.99(0.99,1.00) | 0.186 |
| **TC** |  | |  |  |  |  |  | |  |  |  |  | 1.19(1.08,1.31) | <0.001 | 1.19(1.08,1.32) | 0.001 | 0.88(0.66,1.18) | 0.405 | 0.90(0.67,1.21) | 0.496 | 0.90(0.67,1.21) | 0.498 |
| **TG** |  | |  |  |  |  |  | |  |  |  |  |  |  | 1.00(0.94,1.06) | 0.888 | 1.03(0.97,1.11) | 0.344 | 1.02(0.95,1.10) | 0.605 | 1.02(0.95,1.10) | 0.606 |
| **LDL** |  | |  |  |  |  |  | |  |  |  |  |  |  |  |  | 1.49(1.03,2.14) | 0.034 | 1.42(0.99,2.05) | 0.060 | 1.42(0.98,2.05) | 0.060 |
| **UA** |  | |  |  |  |  |  | |  |  |  |  |  |  |  |  |  |  | 1.00(1.00,1.00) | 0.006 | 1.00(1.00,1.00) | 0.007 |
| **SCr** |  | |  |  |  |  |  | |  |  |  |  |  |  |  |  |  |  |  |  | 1.00(0.99,1.01) | 0.976 |
| **Damp-heat mixed** | 3.16(2.36,4.24) | | <0.001 | 2.43(1.79,3.29) | <0.001 | 1.98(1.45,2.69) | <0.001 | | 1.87(1.33,2.62) | <0.001 | 1.94(1.37,2.75) | <0.001 | 1.97(1.39,2.79) | <0.001 | 1.95(1.38,2.77) | <0.001 | 1.37(0.94,2.00) | 0.104 | 1.37(0.94,2.01) | 0.101 | 1.37(0.94,2.01) | 0.101 |
| **Sex** |  | |  | 0.52(0.43,0.64) | <0.001 | 0.59(0.48,0.71) | <0.001 | | 0.68(0.54,0.85) | 0.001 | 0.68(0.54,0.86) | 0.001 | 0.68(0.54,0.86) | 0.001 | 0.70(0.55,0.88) | 0.003 | 0.87(0.68,1.10) | 0.246 | 0.84(0.63,1.12) | 0.240 | 0.81(0.58,1.15) | 0.238 |
| **Age** |  | |  |  |  | 1.04(1.03,1.05) | <0.001 | | 1.04(1.03,1.05) | <0.001 | 1.04(1.03,1.05) | <0.001 | 1.03(1.02,1.04) | <0.001 | 1.04(1.02,1.05) | <0.001 | 1.03(1.02,1.04) | <0.001 | 1.03(1.02,1.04) | <0.001 | 1.03(1.02,1.04) | <0.001 |
| **BMI** |  | |  |  |  |  |  | | 1.08(1.05,1.12) | <0.001 | 1.08(1.05,1.12) | <0.001 | 1.08(1.04,1.11) | <0.001 | 1.07(1.04,1.11) | <0.001 | 1.06(1.03,1.10) | 0.001 | 1.06(1.03,1.10) | 0.001 | 1.06(1.03,1.10) | 0.001 |
| **SBP** |  | |  |  |  |  |  | |  |  | 1.00(0.99,1.01) | 0.767 | 1.00(0.99,1.01) | 0.964 | 1.00(0.99,1.01) | 0.737 | 1.00(0.99,1.01) | 0.959 | 1.00(0.99,1.01) | 0.964 | 1.00(0.99,1.01) | 0.965 |
| **TC** |  | |  |  |  |  |  | |  |  |  |  | 1.23(1.12,1.36) | <0.001 | 1.19(1.07,1.32) | 0.001 | 0.82(0.60,1.11) | 0.202 | 0.81(0.59,1.11) | 0.189 | 0.81(0.59,1.11) | 0.187 |
| **TG** |  | |  |  |  |  |  | |  |  |  |  |  |  | 1.08(1.04,1.13) | <0.001 | 1.17(1.08,1.26) | <0.001 | 1.17(1.09,1.26) | <0.001 | 1.17(1.08,1.26) | <0.001 |
| **LDL** |  | |  |  |  |  |  | |  |  |  |  |  |  |  |  | 1.58(1.08,2.31) | 0.018 | 1.60(1.09,2.36) | 0.017 | 1.61(1.09,2.37) | 0.016 |
| **UA** |  | |  |  |  |  |  | |  |  |  |  |  |  |  |  |  |  | 1.00(1.00,1.00) | 0.719 | 1.00(1.00,1.00) | 0.782 |
| **SCr** |  | |  |  |  |  |  | |  |  |  |  |  |  |  |  |  |  |  |  | 1.00(0.99,1.01) | 0.722 |
| **Blood stasis mixed** | 3.68(2.74,4.94) | | <0.001 | 3.08(2.28,4.17) | <0.001 | 2.51(1.84,3.40) | <0.001 | | 2.40(1.72,3.35) | <0.001 | 2.53(1.79,3.57) | <0.001 | 2.50(1.77,3.53) | <0.001 | 2.49(1.76,3.52) | <0.001 | 1.74(1.19,2.54) | 0.004 | 1.72(1.18,2.52) | 0.005 | 1.72(1.18,2.52) | 0.005 |
| **Sex** |  | |  | 0.62(0.52,0.74) | <0.001 | 0.66(0.55,0.78) | <0.001 | | 0.76(0.62,0.93) | 0.008 | 0.76(0.61,0.93) | 0.008 | 0.76(0.62,0.94) | 0.010 | 0.77(0.63,0.95) | 0.016 | 0.93(0.75,1.16) | 0.522 | 0.98(0.76,1.27) | 0.885 | 1.01(0.75,1.37) | 0.923 |
| **Age** |  | |  |  |  | 1.03(1.03,1.04) | <0.001 | | 1.03(1.02,1.04) | <0.001 | 1.03(1.02,1.04) | <0.001 | 1.03(1.02,1.04) | <0.001 | 1.03(1.02,1.04) | <0.001 | 1.02(1.01,1.03) | <0.001 | 1.02(1.02,1.03) | <0.001 | 1.02(1.01,1.03) | <0.001 |
| **BMI** |  | |  |  |  |  |  | | 1.07(1.04,1.10) | <0.001 | 1.07(1.04,1.10) | <0.001 | 1.07(1.03,1.10) | <0.001 | 1.06(1.03,1.09) | <0.001 | 1.06(1.02,1.09) | 0.001 | 1.05(1.02,1.09) | 0.002 | 1.05(1.02,1.09) | 0.002 |
| **SBP** |  | |  |  |  |  |  | |  |  | 1.00(0.99,1.01) | 0.634 | 1.00(0.99,1.01) | 0.812 | 1.00(0.99,1.01) | 0.921 | 1.00(0.99,1.01) | 0.570 | 1.00(0.99,1.01) | 0.567 | 1.00(0.99,1.01) | 0.575 |
| **TC** |  | |  |  |  |  |  | |  |  |  |  | 1.21(1.10,1.34) | <0.001 | 1.19(1.07,1.32) | 0.001 | 0.89(0.69,1.16) | 0.390 | 0.90(0.70,1.17) | 0.440 | 0.90(0.69,1.17) | 0.431 |
| **TG** |  | |  |  |  |  |  | |  |  |  |  |  |  | 1.04(0.98,1.10) | 0.179 | 1.10(1.02,1.19) | 0.010 | 1.10(1.02,1.18) | 0.018 | 1.10(1.02,1.18) | 0.017 |
| **LDL** |  | |  |  |  |  |  | |  |  |  |  |  |  |  |  | 1.47(1.07,2.01) | 0.017 | 1.45(1.05,1.98) | 0.023 | 1.45(1.05,1.98) | 0.022 |
| **UA** |  | |  |  |  |  |  | |  |  |  |  |  |  |  |  |  |  | 1.00(1.00,1.00) | 0.452 | 1.00(1.00,1.00) | 0.547 |
| **SCr** |  | |  |  |  |  |  | |  |  |  |  |  |  |  |  |  |  |  |  | 1.00(0.99,1.01) | 0.675 |
| **Yin-deficient mixed** | 3.02(2.17,4.21) | | <0.001 | 2.89(2.06,4.04) | <0.001 | 2.05(1.45,2.89) | <0.001 | | 2.06(1.41,3.01) | <0.001 | 2.14(1.45,3.16) | <0.001 | 2.13(1.44,3.15) | <0.001 | 2.12(1.43,3.14) | <0.001 | 1.34(0.88,2.05) | 0.178 | 1.34(0.88,2.05) | 0.179 | 1.37(0.89,2.09) | 0.152 |
| **Sex** |  | |  | 0.75(0.55,1.03) | 0.074 | 0.85(0.62,1.16) | 0.299 | | 1.07(0.74,1.54) | 0.734 | 1.04(0.71,1.52) | 0.832 | 1.07(0.73,1.58) | 0.715 | 1.15(0.77,1.70) | 0.498 | 1.76(1.15,2.68) | 0.009 | 1.77(1.10,2.83) | 0.018 | 2.34(1.26,4.36) | 0.007 |
| **Age** |  | |  |  |  | 1.06(1.04,1.07) | <0.001 | | 1.05(1.03,1.06) | <0.001 | 1.05(1.03,1.07) | <0.001 | 1.05(1.03,1.07) | <0.001 | 1.05(1.03,1.07) | <0.001 | 1.04(1.02,1.06) | <0.001 | 1.04(1.02,1.06) | <0.001 | 1.04(1.02,1.06) | <0.001 |
| **BMI** |  | |  |  |  |  |  | | 1.12(1.06,1.18) | <0.001 | 1.13(1.07,1.20) | <0.001 | 1.12(1.06,1.19) | <0.001 | 1.11(1.05,1.18) | 0.001 | 1.10(1.03,1.18) | 0.005 | 1.10(1.03,1.18) | 0.007 | 1.09(1.02,1.17) | 0.012 |
| **SBP** |  | |  |  |  |  |  | |  |  | 0.99(0.98,1.00) | 0.120 | 0.99(0.98,1.00) | 0.075 | 0.99(0.98,1.00) | 0.071 | 0.99(0.97,1.00) | 0.076 | 0.99(0.97,1.00) | 0.077 | 0.99(0.98,1.00) | 0.105 |
| **TC** |  | |  |  |  |  |  | |  |  |  |  | 1.33(1.11,1.59) | 0.002 | 1.28(1.06,1.54) | 0.009 | 0.64(0.40,1.01) | 0.056 | 0.64(0.40,1.04) | 0.069 | 0.62(0.38,1.00) | 0.051 |
| **TG** |  | |  |  |  |  |  | |  |  |  |  |  |  | 1.11(1.01,1.22) | 0.026 | 1.32(1.14,1.52) | <0.001 | 1.32(1.14,1.52) | <0.001 | 1.32(1.15,1.53) | <0.001 |
| **LDL** |  | |  |  |  |  |  | |  |  |  |  |  |  |  |  | 2.58(1.42,4.69) | 0.002 | 2.57(1.39,4.75) | 0.003 | 2.62(1.41,4.85) | 0.002 |
| **UA** |  | |  |  |  |  |  | |  |  |  |  |  |  |  |  |  |  | 1.00(1.00,1.00) | 0.959 | 1.00(1.00,1.00) | 0.831 |
| **SCr** |  | |  |  |  |  |  | |  |  |  |  |  |  |  |  |  |  |  |  | 1.01(0.99,1.03) | 0.171 |

BMI, body mass index; SBP, systolic blood pressure; TC, total cholesterol; TG, triglycerides; LDL, low-density lipoprotein cholesterol; UA, uric acid; SCr, serum creatinine.

**Supplementary Table 4. Interaction between TCM constitution and age in the fully adjusted Cox model.**

| **Interaction** | **HR(95%CI)** | P value |
| --- | --- | --- |
| Yang-deficient × age | 1.01(0.96, 1.06) | 0.798 |
| Yin-deficient × age | 1.02(0.98, 1.07) | 0.338 |
| Qi-deficient × age | 0.99(0.95, 1.02) | 0.406 |
| Qi stagnation × age | 1.01(0.96, 1.05) | 0.805 |
| Phlegm-dampness × age | 0.99(0.96, 1.02) | 0.603 |
| Damp-heat × age | 0.98(0.95, 1.01) | 0.138 |
| Blood stasis × age | 0.99(0.95, 1.03) | 0.554 |
| Yang-deficient mixed × age | 0.99(0.94, 1.04) | 0.691 |
| Qi-deficient mixed × age | 0.99(0.96, 1.02) | 0.561 |
| Qi stagnation mixed × age | 0.97(0.95, 1.00) | 0.097 |
| Phlegm-dampness mixed × age | 0.99(0.96, 1.02) | 0.507 |
| Damp-heat mixed × age | 0.98(0.95, 1.01) | 0.125 |
| Blood stasis mixed × age | 0.97(0.94, 1.00) | 0.047 |
| Yin-deficient mixed × age | 0.97(0.94, 1.01) | 0.138 |

Age was treated as a continuous variable, and statistical significance was assessed using the Wald test.

HR, hazard ratio; 95% CI, 95% confidence interval.

**Supplementary Table 5. Association between TCM constitution and risk of hyperglycemia in age-stratified analyses (18-39, ≥ 40 years).**

| **TCM constitution** | **N** | **Crude HR(95%CI)** | ***P*** value | **N** | **Full-adjusted HR(95%CI)** | ***P*** value |
| --- | --- | --- | --- | --- | --- | --- |
| **18-39 years** | | | | | | |
| Balanced | 34 | Ref. | Ref. | 22 | Ref. | Ref. |
| Single biased constitution |  |  |  |  |  |  |
| Yang-deficient | 6 | / | / | 4 | / | / |
| Yin-deficient | 12 | / | / | 9 | / | / |
| Qi-deficient | 39 | 1.67(1.05,2.65) | 0.030 | 29 | 1.08(0.60,1.95) | 0.796 |
| Qi stagnation | 36 | 4.14(2.45,6.98) | <0.001 | 30 | 2.01(1.01,4.03) | 0.048 |
| Phlegm-dampness | 120 | 3.19(2.17,4.68) | <0.001 | 97 | 1.65(0.98,2.77) | 0.059 |
| Damp-heat | 355 | 2.94(2.06,4.20) | <0.001 | 293 | 1.66(1.06,2.60) | 0.026 |
| Blood stasis | 37 | 5.13(2.98,8.84) | <0.001 | 32 | 2.35(1.16,4.76) | 0.018 |
| Mixed biased constitution |  |  |  |  |  |  |
| Yang-deficient mixed | 15 | / | / | 15 | / | / |
| Qi-deficient mixed | 100 | 2.79(1.88,4.14) | <0.001 | 81 | 1.37(0.82,2.29) | 0.237 |
| Qi stagnation mixed | 149 | 4.42(2.99,6.55) | <0.001 | 134 | 1.80(1.07,3.04) | 0.027 |
| Phlegm-dampness mixed | 249 | 3.51(2.43,5.07) | <0.001 | 209 | 1.51(0.93,2.43) | 0.093 |
| Damp-heat mixed | 211 | 3.26(2.25,4.71) | <0.001 | 183 | 1.48(0.93,2.35) | 0.102 |
| Blood stasis mixed | 243 | 4.09(2.82,5.93) | <0.001 | 212 | 1.96(1.23,3.13) | 0.005 |
| Yin-deficient mixed | 53 | 2.82(1.81,4.41) | <0.001 | 48 | 1.47(0.85,2.53) | 0.167 |
| **≥ 40 years** | | | | | | |
| Balanced | 17 | Ref. | Ref. | 10 | Ref. | Ref. |
| Single biased constitution |  |  |  |  |  |  |
| Yang-deficient | 16 | 1.13(0.57,2.25) | 0.721 | 15 | 2.83(0.85,9.44) | 0.091 |
| Yin-deficient | 47 | 0.98(0.56,1.70) | 0.932 | 38 | 0.69(0.28,1.71) | 0.425 |
| Qi-deficient | 59 | 0.90(0.52,1.54) | 0.688 | 47 | 0.81(0.38,1.71) | 0.584 |
| Qi stagnation | 48 | 1.23(0.70,2.16) | 0.466 | 39 | 2.11(0.88,5.07) | 0.095 |
| Phlegm-dampness | 116 | 1.45(0.87,2.41) | 0.154 | 92 | 1.37(0.67,2.78) | 0.386 |
| Damp-heat | 327 | 1.17(0.72,1.90) | 0.539 | 264 | 1.06(0.56,2.02) | 0.861 |
| Blood stasis | 56 | 1.43(0.82,2.48) | 0.203 | 51 | 1.51(0.70,3.26) | 0.296 |
| Mixed biased constitution |  |  |  |  |  |  |
| Yang-deficient mixed | 20 | 1.23(0.64,2.37) | 0.531 | 12 | / | / |
| Qi-deficient mixed | 102 | 1.14(0.68,1.91) | 0.616 | 79 | 0.90(0.45,1.79) | 0.759 |
| Qi stagnation mixed | 161 | 1.50(0.91,2.48) | 0.113 | 125 | 1.47(0.75,2.90) | 0.267 |
| Phlegm-dampness mixed | 235 | 1.28(0.78,2.10) | 0.324 | 182 | 1.00(0.52,1.93) | 0.998 |
| Damp-heat mixed | 231 | 1.18(0.72,1.94) | 0.502 | 170 | 1.02(0.53,1.99) | 0.943 |
| Blood stasis mixed | 256 | 1.26(0.77,2.07) | 0.350 | 198 | 1.19(0.62,2.30) | 0.604 |
| Yin-deficient mixed | 77 | 1.24(0.73,2.10) | 0.420 | 60 | 1.00(0.47,2.11) | 0.991 |

The symbol "/" represented that the bias constitution was excluded, because of the sample size (n≤15).

HR, hazard ratio; 95% CI, 95% confidence interval.

**Supplementary Table 6. Association between TCM constitution and risk of hyperglycemia in follow-up-stratified analyses (<24 months, ≥ 24 months).**

| **TCM constitution** | **N** | **Crude HR(95%CI)** | ***P* value** | **N** | **Full-adjusted HR(95%CI)** | ***P* value** |
| --- | --- | --- | --- | --- | --- | --- |
| **Follow-up duration < 24 months** | | | | | | |
| Balanced | 23 | Ref. | Ref. | 11 | Ref. | Ref. |
| Single biased constitution |  |  |  |  |  |  |
| Yang-deficient | 7 | / | / | 6 | / | / |
| Yin-deficient | 26 | 1.09(0.62,1.91) | 0.767 | 18 | 0.72(0.29,1.79) | 0.480 |
| Qi-deficient | 40 | 1.44(0.86,2.42) | 0.166 | 25 | 1.04(0.44,2.45) | 0.934 |
| Qi stagnation | 34 | 1.10(0.64,1.89) | 0.722 | 27 | 0.57(0.22,1.45) | 0.235 |
| Phlegm-dampness | 124 | 1.26(0.81,1.97) | 0.303 | 98 | 1.08(0.56,2.07) | 0.828 |
| Damp-heat | 286 | 1.12(0.73,1.72) | 0.592 | 220 | 0.91(0.48,1.70) | 0.765 |
| Blood stasis | 35 | 1.21(0.71,2.07) | 0.485 | 30 | 0.53(0.22,1.31) | 0.168 |
| Mixed biased constitution |  |  |  |  |  |  |
| Yang-deficient mixed | 14 | / | / | 9 | / | / |
| Qi-deficient mixed | 78 | 1.12(0.70,1.80) | 0.627 | 64 | 0.81(0.40,1.65) | 0.557 |
| Qi stagnation mixed | 116 | 1.30(0.83,2.05) | 0.249 | 95 | 0.75(0.37,1.51) | 0.419 |
| Phlegm-dampness mixed | 175 | 1.23(0.80,1.91) | 0.350 | 138 | 0.94(0.48,1.83) | 0.859 |
| Damp-heat mixed | 154 | 1.22(0.79,1.90) | 0.369 | 124 | 1.05(0.54,2.03) | 0.884 |
| Blood stasis mixed | 199 | 1.25(0.81,1.93) | 0.312 | 165 | 1.04(0.55,1.96) | 0.914 |
| Yin-deficient mixed | 46 | 1.06(0.63,1.77) | 0.830 | 39 | 0.87(0.41,1.83) | 0.710 |
| **Follow-up duration ≥ 24 months** | | | | | | |
| Balanced | 28 | Ref. | Ref. | 21 | Ref. | Ref. |
| Single biased constitution |  |  |  |  |  |  |
| Yang-deficient | 15 | / | / | 13 | / | / |
| Yin-deficient | 33 | 2.27(1.37,3.77) | 0.002 | 29 | 1.00(0.51,1.96) | 0.999 |
| Qi-deficient | 58 | 2.30(1.46,3.61) | <0.001 | 51 | 0.98(0.56,1.69) | 0.930 |
| Qi stagnation | 50 | 6.21(3.69,10.45) | <0.001 | 42 | 2.65(1.32,5.29) | 0.006 |
| Phlegm-dampness | 112 | 3.59(2.36,5.44) | <0.001 | 91 | 1.40(0.80,2.45) | 0.235 |
| Damp-heat | 396 | 3.65(2.48,5.37) | <0.001 | 337 | 1.60(1.02,2.52) | 0.042 |
| Blood stasis | 58 | 8.13(4.87,13.57) | <0.001 | 53 | 3.35(1.75,6.39) | <0.001 |
| Mixed biased constitution |  |  |  |  |  |  |
| Yang-deficient mixed | 21 | 4.45(2.46,8.04) | <0.001 | 18 | 3.05(1.36,6.85) | 0.007 |
| Qi-deficient mixed | 124 | 3.63(2.04,5.50) | <0.001 | 96 | 1.22(0.73,2.03) | 0.446 |
| Qi stagnation mixed | 194 | 6.29(4.16,9.52) | <0.001 | 164 | 2.32(1.39,3.90) | 0.001 |
| Phlegm-dampness mixed | 309 | 4.76(3.21,7.05) | <0.001 | 253 | 1.54(0.96,2.47) | 0.070 |
| Damp-heat mixed | 288 | 4.51(3.05,6.68) | <0.001 | 229 | 1.48(0.92,2.37) | 0.104 |
| Blood stasis mixed | 300 | 5.30(3.57,7.86) | <0.001 | 245 | 1.95(1.22,3.14) | 0.006 |
| Yin-deficient mixed | 84 | 4.51(2.91,6.98) | <0.001 | 69 | 1.61(0.95,2.76) | 0.079 |

The symbol "/" represented that the bias constitution was excluded, because of the sample size (n≤15).

HR, hazard ratio; 95% CI, 95% confidence interval.

**Supplementary Table 7. Association between TCM constitution and risk of hyperglycemia in follow-up-stratified analyses (6-30 months, ≥30 months).**

| **TCM constitution** | **N** | **Crude HR(95%CI)** | ***P* value** | **N** | **Full-adjusted HR(95%CI)** | ***P* value** |
| --- | --- | --- | --- | --- | --- | --- |
| **6 months ≤ Follow-up duration < 30 months** | | | | | | |
| Balanced | 27 | Ref. | Ref. | 15 | Ref. | Ref. |
| Single biased constitution |  |  |  |  |  |  |
| Yang-deficient | 10 | / | / | 8 | / | / |
| Yin-deficient | 31 | 1.13(0.67,1.90) | 0.655 | 22 | 1.30(0.60,2.80) | 0.508 |
| Qi-deficient | 51 | 1.43(0.90,2.29) | 0.132 | 36 | 1.35(0.69,2.64) | 0.378 |
| Qi stagnation | 49 | 1.35(0.84,2.16) | 0.216 | 40 | 1.14(0.56,2.33) | 0.715 |
| Phlegm-dampness | 144 | 1.59(1.05,2.40) | 0.027 | 116 | 1.49(0.84,2.67) | 0.173 |
| Damp-heat | 414 | 1.35(0.91,1.99) | 0.133 | 328 | 1.28(0.75,2.18) | 0.368 |
| Blood stasis | 55 | 1.43(0.90,2.28) | 0.128 | 50 | 1.17(0.59,2.33) | 0.648 |
| Mixed biased constitution |  |  |  |  |  |  |
| Yang-deficient mixed | 20 | 1.36(0.76,2.43) | 0.300 | 14 | / | / |
| Qi-deficient mixed | 113 | 1.32(0.86,2.01) | 0.201 | 91 | 1.35(0.75,2.45) | 0.319 |
| Qi stagnation mixed | 168 | 1.60(1.07,2.41) | 0.024 | 142 | 1.37(0.77,2.43) | 0.289 |
| Phlegm-dampness mixed | 239 | 1.36(0.91,2.03) | 0.132 | 194 | 1.33(0.75,2.33) | 0.327 |
| Damp-heat mixed | 219 | 1.40(0.94,2.08) | 0.103 | 182 | 1.32(0.76,2.31) | 0.326 |
| Blood stasis mixed | 282 | 1.46(0.98,2.17) | 0.060 | 237 | 1.38(0.80,2.38) | 0.240 |
| Yin-deficient mixed | 65 | 1.23(0.78,1.93) | 0.370 | 56 | 1.11(0.60,2.06) | 0.748 |
| **Follow-up duration ≥ 30 months** | | | | | | |
| Balanced | 24 | Ref. | Ref. | 17 | Ref. | Ref. |
| Single biased constitution |  |  |  |  |  |  |
| Yang-deficient | 12 | / | / | 11 | / | / |
| Yin-deficient | 28 | 2.32(1.34,4.02) | 0.003 | 25 | 1.09(0.52,2.31) | 0.813 |
| Qi-deficient | 47 | 2.25(1.37,3.69) | 0.001 | 40 | 1.04(0.55,1.96) | 0.907 |
| Qi stagnation | 35 | 6.44(3.59,11.56) | <0.001 | 29 | 3.48(1.51,8.02) | 0.003 |
| Phlegm-dampness | 92 | 3.64(2.31,5.72) | <0.001 | 73 | 1.69(0.91,3.14) | 0.094 |
| Damp-heat | 267 | 3.34(2.19,5.08) | <0.001 | 229 | 1.61(0.97,2.69) | 0.065 |
| Blood stasis | 38 | 8.31(4.65,14.85) | <0.001 | 33 | 4.45(2.06,9.64) | <0.001 |
| Mixed biased constitution |  |  |  |  |  |  |
| Yang-deficient mixed | 15 | / | / | 13 | / | / |
| Qi-deficient mixed | 89 | 3.40(2.16,5.37) | <0.001 | 69 | 1.18(0.66,2.10) | 0.582 |
| Qi stagnation mixed | 142 | 6.36(4.05,9.99) | <0.001 | 117 | 2.61(1.45,4.68) | 0.001 |
| Phlegm-dampness mixed | 243 | 4.87(3.18,7.46) | <0.001 | 195 | 1.61(0.95,2.72) | 0.074 |
| Damp-heat mixed | 221 | 4.48(2.93,6.87) | <0.001 | 169 | 1.69(0.99,2.87) | 0.054 |
| Blood stasis mixed | 217 | 5.25(3.41,8.06) | <0.001 | 173 | 1.99(1.17,3.40) | 0.011 |
| Yin-deficient mixed | 65 | 4.54(2.81,7.33) | <0.001 | 52 | 1.73(0.94,3.21) | 0.080 |

The symbol "/" represented that the bias constitution was excluded, because of the sample size (n≤15).

HR, hazard ratio; 95% CI, 95% confidence interval.

**Supplementary Table 8. Sample sizes for each TCM constitution group before and after PSM.**

| **TCM constitution** | **Unmatched** | | **Matched** | |
| --- | --- | --- | --- | --- |
|  | **Balanced** | **Bias** | **Balanced** | **Bias** |
| Yang-deficient | 362 | 71 | 47 | 47 |
| Yin-deficient | 362 | 252 | 162 | 162 |
| Qi-deficient | 362 | 384 | 233 | 233 |
| Qi stagnation | 362 | 294 | 126 | 126 |
| Phlegm-dampness | 362 | 870 | 287 | 287 |
| Damp-heat | 362 | 2,521 | 330 | 330 |
| Blood stasis | 362 | 294 | 142 | 142 |
| Yang-deficient mixed | 362 | 126 | 75 | 75 |
| Qi-deficient mixed | 362 | 737 | 272 | 272 |
| Qi stagnation mixed | 362 | 898 | 251 | 251 |
| Phlegm-dampness mixed | 362 | 1621 | 311 | 311 |
| Damp-heat mixed | 362 | 1410 | 322 | 322 |
| Blood stasis mixed | 362 | 1597 | 324 | 324 |
| Yin-deficient mixed | 362 | 437 | 248 | 248 |

Each biased constitution type was 1:1 matched to balanced constitution participants using nearest-neighbor matching (caliper = 0.2 SD of logit PS), based on age, sex, and BMI.

**Supplementary Table 9. Association between TCM constitution and risk of hyperglycemia after PSM.**

| **TCM constitution** | **Balanced(N) / Biased(N)** | **Crude HR(95%CI)** | ***P* value** | **Balanced(N) / Biased(N)** | **Full-adjusted HR(95%CI)** | ***P* value** |
| --- | --- | --- | --- | --- | --- | --- |
| Balanced |  | Ref. | Ref. |  | Ref. | Ref. |
| Single biased constitution |  |  |  |  |  |  |
| Yang-deficient | 12/12 | 1.02(0.45,2.30) | 0.962 | 11/12 | 1.75(0.61,5.08) | 0.300 |
| Yin-deficient | 32/29 | 0.82(0.50,1.36) | 0.454 | 20/23 | 0.76(0.40,1.44) | 0.392 |
| Qi-deficient | 39/36 | 0.86(0.54,1.35) | 0.500 | 31/30 | 0.84(0.50,1.41) | 0.500 |
| Qi stagnation | 25/28 | 2.07(1.15,3.74) | 0.015 | 21/27 | 1.38(0.68,2.80) | 0.377 |
| Phlegm-dampness | 43/50 | 2.05(1.35,3.10) | 0.001 | 32/42 | 1.91(1.15,3.18) | 0.012 |
| Damp-heat | 44/32 | 0.91(0.57,1.44) | 0.675 | 32/30 | 1.08(0.60,1.94) | 0.789 |
| Blood stasis | 34/35 | 1.73(1.04,2.86) | 0.034 | 27/33 | 1.89(1.04,3.42) | 0.036 |
| Mixed biased constitution |  |  |  |  |  |  |
| Yang-deficient mixed | 15/17 | 1.42(0.69,2.89) | 0.341 | 14/17 | 1.74(0.73,4.11) | 0.211 |
| Qi-deficient mixed | 42/50 | 1.40(0.92,2.11) | 0.112 | 31/48 | 1.37(0.82,2.28) | 0.232 |
| Qi stagnation mixed | 40/52 | 2.09(1.35,3.21) | 0.001 | 30/51 | 1.71(1.00,2.90) | 0.049 |
| Phlegm-dampness mixed | 44/55 | 1.76(1.17,2.65) | 0.006 | 32/53 | 1.39(0.82,2.36) | 0.226 |
| Damp-heat mixed | 44/49 | 1.37(0.91,2.07) | 0.130 | 32/47 | 1.19(0.71,1.99) | 0.504 |
| Blood stasis mixed | 44/56 | 1.93(1.28,2.92) | 0.002 | 32/52 | 1.42(0.85,2.39) | 0.183 |
| Yin-deficient mixed | 38/56 | 2.02(1.32,3.08) | 0.001 | 27/52 | 1.37(0.84,2.24) | 0.201 |

SBP, TC, TG, LDL-C, UA, and SCr were adjusted.

HR, hazard ratio; 95% CI, 95% confidence interval.

**Supplementary Table 10. Distribution of TCM constitution in population stratified by newly onset prediabetes and diabetes.**

| **TCM constitution** | **Newly onset of hyperglycemia** | | | |
| --- | --- | --- | --- | --- |
|  | **Prediabetes (n=2,332)** | ***P* value** | **Diabetes(n=44)** | ***P* value** |
| Balanced constitution (n, %) | 51(2.19) | <0.001 | 0(0.00) | 0.124 |
| Single biased constitution (n, %) |  |  |  |  |
| Yang-deficient | 22(30.14) | 0.001 | 0(0.00) | / |
| Yin-deficient | 59(53.64) | 0.003 | 0(0.00) | / |
| Qi-deficient | 97(65.54) | <0.001 | 1(100.00) | 0.297 |
| Qi stagnation | 82(61.65) | <0.001 | 2(100.00) | 0.086 |
| Phlegm-dampness | 230(81.85) | <0.001 | 6(100.00) | 0.087 |
| Damp-heat | 668(92.91) | <0.001 | 14(100.00) | 0.124 |
| Blood stasis | 92(64.34) | <0.001 | 1(100.00) | 0.214 |
| Mixed biased constitution (n, %) |  |  |  |  |
| Yang-deficient mixed | 34(40.00) | 0.001 | 1(100.00) | 0.066 |
| Qi-deficient mixed | 199(79.60) | <0.001 | 3(100.00) | 0.187 |
| Qi stagnation mixed | 304(85.63) | <0.001 | 6(100.00) | 0.075 |
| Phlegm-dampness mixed | 475(90.30) | <0.001 | 9(100.00) | 0.117 |
| Damp-heat mixed | 433(89.46) | <0.001 | 9(100.00) | 0.089 |
| Blood stasis mixed | 491(90.59) | <0.001 | 8(100.00) | 0.133 |
| Yin-deficient mixed | 126(71.19) | <0.001 | 4(100.00) | 0.045 |

Intrinsic/Allergic constitution was excluded because of the sample size (n=1). Fisher's precision probability tests were used for those with sample size lower than 5.

**Supplementary Table 11. Association between TCM constitution and risk of newly onset prediabetes.**

| **TCM constitution** | **Crude HR(95%CI)** | ***P* value** | **Full-adjusted HR(95%CI)** | ***P* value** |
| --- | --- | --- | --- | --- |
| Balanced constitution | Ref. | Ref. | Ref. | Ref. |
| Single biased constitution |  |  |  |  |
| Yang-deficient | 2.66(1.61,4.40) | <0.001 | 2.27(1.13,4.57) | 0.021 |
| Yin-deficient | 1.94(1.33,2.83) | 0.001 | 0.93(0.55,1.56) | 0.783 |
| Qi-deficient | 1.93(1.37,2.72) | <0.001 | 0.98(0.63,1.53) | 0.924 |
| Qi stagnation | 3.45(2.36,5.05) | <0.001 | 1.81(1.06,3.07) | 0.029 |
| Phlegm-dampness | 3.07(2.26,4.17) | <0.001 | 1.63(1.08,2.46) | 0.020 |
| Damp-heat | 2.73(2.05,3.64) | <0.001 | 1.47(1.02,2.12) | 0.038 |
| Blood stasis | 4.22(2.89,6.16) | <0.001 | 2.28(1.37,3.79) | 0.001 |
| Mixed biased constitution |  |  |  |  |
| Yang-deficient mixed | 2.77(1.77,4.35) | <0.001 | 1.84(0.98,3.44) | 0.056 |
| Qi-deficient mixed | 2.63(1.93,3.60) | <0.001 | 1.25(0.83,1.88) | 0.291 |
| Qi stagnation mixed | 4.05(2.97,5.52) | <0.001 | 1.87(1.24,2.82) | 0.003 |
| Phlegm-dampness mixed | 3.23(2.40,4.33) | <0.001 | 1.41(0.97,2.07) | 0.075 |
| Damp-heat mixed | 3.11(2.32,4.17) | <0.001 | 1.37(0.94,2.01) | 0.103 |
| Blood stasis mixed | 3.63(2.70,4.87) | <0.001 | 1.72(1.17,2.51) | 0.006 |
| Yin-deficient mixed | 2.95(2.11,4.12) | <0.001 | 1.37(0.89,2.09) | 0.152 |

Age, sex, BMI, SBP, TC, TG, LDL-C, UA, and SCr were adjusted.

HR, hazard ratio; 95% CI, 95% confidence interval.
